# Functional Traits for Carbon Access in Macrophytes

**DOI:** 10.1371/journal.pone.0159062

**Published:** 2016-07-14

**Authors:** Courtney C. Stepien, Catherine A. Pfister, J. Timothy Wootton

**Affiliations:** 1 Committee on Evolutionary Biology, University of Chicago, Chicago, IL, United States of America; 2 Department of Ecology and Evolution, University of Chicago, Chicago, IL, United States of America; University of Connecticut, UNITED STATES

## Abstract

Understanding functional trait distributions among organisms can inform impacts on and responses to environmental change. In marine systems, only 1% of dissolved inorganic carbon in seawater exists as CO_2_. Thus the majority of marine macrophytes not only passively access CO_2_ for photosynthesis, but also actively transport CO_2_ and the more common bicarbonate (HCO_3_^-^, 92% of seawater dissolved inorganic carbon) into their cells. Because species with these carbon concentrating mechanisms (CCMs) are non-randomly distributed in ecosystems, we ask whether there is a phylogenetic pattern to the distribution of CCMs among algal species. To determine macrophyte traits that influence carbon uptake, we assessed 40 common macrophyte species from the rocky intertidal community of the Northeast Pacific Ocean to a) query whether macrophytes have a CCM and b) determine the evolutionary history of CCMs, using ancestral state reconstructions and stochastic character mapping based on previously published data. Thirty-two species not only depleted CO_2_, but also concentrated and depleted HCO_3_^-^, indicative of a CCM. While analysis of CCMs as a continuous trait in 30 families within Phylum Rhodophyta showed a significant phylogenetic signal under a Brownian motion model, analysis of CCMs as a discrete trait (presence or absence) indicated that red algal families are more divergent than expected in their CCM presence or absence; CCMs are a labile trait within the Rhodophyta. In contrast, CCMs were present in each of 18 Ochrophyta families surveyed, indicating that CCMs are highly conserved in the brown algae. The trait of CCM presence or absence was largely conserved within Families. Fifteen of 23 species tested also changed the seawater buffering capacity, or Total Alkalinity (TA), shifting DIC composition towards increasing concentrations of HCO_3_^-^ and CO_2_ for photosynthesis. Manipulating the external TA of the local environment may influence carbon availability in boundary layers and areas of low water mixing, offering an additional mechanism to increase CO_2_ availability.

## Introduction

Traits determine both the environmental impacts on, and environmental effects of, organisms, processes that ultimately shape evolution through natural selection. Hence, understanding trait distributions among species can help to anticipate ecological effects of environmental change. Most studies focus on morphological traits, or other traits that are readily measured in museum collections or laboratories. Though often more difficult to measure than morphological traits, functional traits offer direct insight into ecologically important differences among species that may control both evolutionary history and the scope for future adaptation.

Changes in the global carbon cycle arising from anthropogenic CO_2_ release to the atmosphere may both affect, and be affected by, many species within ecosystems [1–3]. Unlike terrestrial situations, where plants interact with inorganic carbon solely as CO_2_, which has a high diffusion rate in air, aquatic plants exist in a medium with low CO_2_ diffusion and in which dissolved inorganic carbon (DIC) occurs in several forms. In some marine environments, photosynthesis can be carbon-limited owing to slow diffusion of CO_2_ in water, local depletion of dissolved inorganic carbon (DIC), and pH-dependent shifts in the concentrations of DIC forms in seawater. As phototrophs deplete DIC, the relative equilibrium concentrations of DIC remaining (bicarbonate: HCO_3_^-^, carbon dioxide: CO_2_, and carbonate: CO_3_^2-^) change and drive seawater to higher pH. Alternately, as dissolved inorganic carbon increases in seawater, CO_2_ and HCO_3_^-^ increase in concentration, while CO_3_^2-^ declines and pH drops (S1 Fig). Thus, pH provides a proxy for DIC proportions. At ambient seawater pH of roughly 8.0, HCO_3_^-^ comprises 92% of DIC in the surface ocean, with CO_2_ concentration at 1%. At pH > 9.0, however, CO_2_ concentration approaches zero. In addition to relying on passive diffusion of CO_2_, the majority of marine macroalgal species can utilize a carbon concentrating mechanism (CCM). Plants with CCMs can actively transport HCO_3_^-^ or CO_2_ into plant cells, thereby increasing photosynthetic rates under high competition for DIC [4]. CCMs are hypothesized to have evolved in response to historically carbon-limited environments [5] and occur over taxonomically and spatially diverse species [6,7] with differing underlying physiological mechanisms [8].

Marine and terrestrial phototrophs alike employ CCMs to increase inorganic carbon availability (for example, C4 and CAM plants in terrestrial ecosystems), but they do so under different conditions. In coastal marine systems, variation in upwelling, temperature, pH, salinity, and terrestrial inputs can all influence local seawater DIC availability and relative proportions of dissolved CO_2_, HCO_3_^-^ and CO_3_^2-^ [9], while intertidal macroalgae experience the added complexity of daily exposure to atmospheric CO_2_. Because CO_2_ diffuses more slowly in water, DIC availability in boundary layers surrounding macrophytes may become locally depleted as organisms take up CO_2_ [10,11], increasing pH during hours of peak photosynthesis [1]. Taken together, environmental variation and biotic interactions create variable CO_2_ concentrations in coastal marine systems.

Marine macrophytes with CCMs are distributed non-randomly along global and local environmental gradients. Species sampled in warmer climates have tissue δ^13^C values more indicative of bicarbonate use than those in cooler environments, implying potential carbon-limiting environments in the tropics [12,13]. There also appears to be a latitudinal trend in CCM prevalence based on δ^13^C trends, where a greater proportion of species near the poles have isotope signatures indicative of CCM absence [13]. Vertical depth trends have also been observed along the coastal emersion gradient from the intertidal to the subtidal, with decreasing CCM frequency at lower depths [13,14] potentially caused by light limitation [15]. Macroalgae that depend on passive diffusion of CO_2_ may directly benefit from elevated CO_2_ arising from anthropogenic activities [16], whereas those that use HCO_3_^-^ or create conditions that favor increased CO_2_ diffusion may be at a disadvantage because these mechanisms can carry energetic costs [6]. In phytoplankton for example, elevated CO_2_ conditions may shift dominance among species due to differential abilities of species to concentrate DIC for photosynthesis [17]. Here we evaluate the functional trait of carbon access, as well as how this macrophyte trait may result in a positive effect on further carbon acquisition.

In addition to affecting seawater pH through changes in DIC arising from photosynthesis and respiration, there is evidence that organisms alter the expected pH-DIC relationship by changing the buffering capacity of seawater [18], which may further feed back to local DIC availability. Total alkalinity (TA) reflects the acid buffering capacity of water and comprises all compounds that minimize seawater pH change. While DIC makes up the majority of ambient seawater total alkalinity, other compounds such as borates and organic compounds comprise the rest [19]. Under the expected relationship between DIC and pH—in which TA is held constant—pH increases as DIC decreases. Whereas photosynthesis and respiration do not change TA [19], a net uptake of negative ions by algae, such as OH^-^ [20], or a release of positively charged compounds that bind to proton acceptors may result in drops in TA. As TA decreases, more H^+^ remains in solution causing pH decline, and the proportions of photosynthetically useful HCO_3_^-^ and CO_2_ increase. Spatio-temporal variation in TA occurs in reefs from coral calcification [21,22]. Nutrient exchange by phytoplankton, microbes and microalgae can also affect TA [19,23,24]. By influencing seawater pH without directly acting on DIC, TA shifts present a distinct pathway from active carbon transport or proton pumps for modifying DIC availability.

Here we use pH drift assays and carbon isotope analyses to assess the status and variability of marine macrophyte CCMs from the Northeast Pacific. pH drift assays measure DIC depletion by macrophytes in sealed containers under continuous photosynthesis, and show high agreement with CCM designations based on time-integrated macrophyte tissue carbon isotope values (see Results in [13, 14]). Tissue δ^13^C between -10 and 0‰ are expected in macrophytes that only use HCO_3_^-^, while exclusive CO_2_ use would result in a depleted δ^13^C of -30‰. Thus, δ^13^C values are also used as an indicator of CCM status [4,9,12]. We also supplement pH assays with TA measurements and show that macrophytes vary in HCO_3_^-^ use, with some species unexpectedly altering TA to a degree not fully accounted for by HCO_3_^-^ uptake or calcification. Macrophytes may lower total alkalinity through means other than DIC depletion or calcification, increasing access to DIC for photosynthesis in the process, and potentially influencing species' local access to DIC. We then use phylogenetic analyses of CCMs to ask how CCM presence has evolved in the red and brown algal lineages.

## Materials and Methods

### pH and total alkalinity assays

We assayed how 39 species of marine macroalgae (Rhodophyta: Florideophyceae and Bangiaceae, Chlorophyta: Ulvophyceae and Ochrophyta: Phaeophyceae) and one surfgrass species (Tracheophyta: Monocots) alter seawater pH through DIC depletion using a pH drift assay (modified from [7,25,26]). Assays included five coralline red algal species (three upright and two crustose species), which also affect water chemistry through calcification. Species represented conspicuous taxa from the near-subtidal to high intertidal on emergent rocky benches at Shi Shi Beach, Olympic National Park, Washington, USA (48.28°N, 124.68°W) and Slip Point, Clallam Bay, Washington, USA (48.26°N, 124.25°W), collected from 21 June– 13 July 2013. The National Park Service (Olympic NP) provided access at southern Shi Shi Beach, and the Makah Tribal Nation provided access to private property on Tatoosh Island and northern Shi Shi Beach. Local vertical range (low, mid or high intertidal zone) was noted; when species spanned multiple zones, specimens were collected across their full vertical range to determine within-species variance. Based on specimen availability, 2–13 replicates per species were assayed. Specimens were rinsed, cleaned of visible epiphytes and stored at 7°C for 12–48 hours, then rinsed twice in sterilized seawater, patted dry and separated into 4 g amounts (wet mass). Due to differing individual mass among and within species, some replicates contained multiple individuals, some contained a single individual, and some contained a partial individual. Where cut individuals were used (*Saccharina groendlandica*, *S*. *sessilis*, *Codium fragile*, *and Osmundea spectabilis)*, we always used a mix of whole individuals and partial individual replicates—with each partial replicate from a unique individual—to ensure that exudates from cut individuals did not overwhelm the signal of whole individuals, with the exception of one larger kelp species (*Alaria marginata*) for which small specimens were unavailable. A ratio of 4 g macrophyte to 125 mL seawater (0.032 g/mL) in our assay fell within the range of biomass-to-seawater-volume ratios previously reported in pH assay studies (0.007–0.143 g/mL) [7,18,27], while allowing us to use entire individuals and minimize self-shading. Although calcifying species differ in their photosynthetic biomass to total biomass ratios due to their calcium carbonate skeletons (75–80% inorganic content, versus 10–50% for fleshy species [28]), we applied the same 4 g mass treatment to investigate if calcifying species raise pH* > 9.0 and how they might influence seawater TA.

We collected seawater 150 m off Sekiu, Washington, USA (48.26°N, -124.30°W), and measured seawater salinity, temperature and pH. To minimize the influence of microbes on pH, we filtered seawater through a polypropylene filter net (Aquatic Ecosystems) and a Whatman GF/C filter (1.2 μm pore size), and then UV-treated water (TMC V2 8 Watt) for 24 hours at a 450 lph flow rate. Water was allowed to equilibrate to outdoor atmospheric conditions and used within 48 hours of sterilization. Because fresh seawater was collected and processed for each assay, we tested whether 5 of the 11 seawater batches differed in nutrient profiles. Phosphate, silica, nitrate, nitrite and ammonium concentrations were quantified at the University of Washington Marine Chemistry Lab following filtration and UV treatment. One seawater batch was also assayed for initial TA.

Macrophytes were placed with sterilized seawater in 125 mL sealed clear polystyrene containers and incubated under full-spectrum light alongside seawater controls in a 12°C water bath, a temperature reflecting mean June-July sea surface temperature near collection sites [1]. Macrophytes were exposed to 286 ± 5 μmol/m^2^s photosynthetically active radiation under full-spectrum Marine White Lights (TMC Aquaray Aquabeam 1000 HD Ultra) for 24 hours, an incubation length comparable to other pH assay studies, which ensured ample time for macrophytes to reach a steady-state pH* [7,27]. Jars were placed on an 18 rpm shaker rack to disrupt boundary layer formation. Five species, typically in six replicate containers, were run with six seawater controls without macroalgae in each trial. Specimens and controls were randomized in water bath position and consequently also randomized in measurement order for pH and TA, minimizing potential effects of machine drift. Species replicates were also run across multiple trials using different seawater batches to determine if different seawater stocks significantly influenced the assay.

We recorded seawater pH of each jar at 0, 12 and 24 hours using an IntelliCAL pH probe (PCH101) and HACH meter (HQ40d) calibrated with NBS pH 7.0 and 10.0 standards before each incubation (Thermo-Fisher Scientific). As species deplete dissolved CO_2_, DIC decreases, which causes an increase in pH. At pH = 9.0, CO_2_ concentration is too low (< 1 μmol/kg seawater) to be biologically accessible for the majority of macroalgae and DIC depletion stops. However, if species have a CCM and can access HCO_3_^-^, DIC is further depleted and pH increases beyond 9.0 [25]. The upper boundary of pH drift—pH*—reflects the minimum DIC concentration that a species can access (sensu [29]). Regardless of whether pH* is a result of DIC-independent physiological limitations caused by high pH [30] or of DIC-dependent limitations because DIC is too low, pH* represents a concentration beyond which a species cannot further deplete DIC [27]. In preliminary 32-hour assays, 11 of 12 species reached pH* within 12–24 hours, indicating that 24 hours was a sufficient incubation length. While *Neorhodomela larix* pH* did not stabilize within 32 hours of incubation, seawater pH reached 9.63 by 24 hours, so the CCM categorization of *N*. *larix* was unaffected. The rank order of pH of seawater incubated with different macrophyte species after 12 hours mirrored their order after 24 hours, indicating that any incubation effects accumulating in the last 12 hours did not alter the relative effects of species on seawater.

To determine if macrophytes had a lasting effect on seawater pH beyond depleting DIC, we unsealed containers post-incubation, removed macrophytes and waited a further 24 hours for 40 mL of seawater to re-equilibrate with outdoor atmospheric CO_2_ in the 12°C water bath. pH was then remeasured as the pH of equilibration with the atmosphere (pH_e_). To make pH_e_ values comparable across assays from different seawater batches and to control for variations in ambient atmospheric pCO_2_ through time, pH_e_ was reported as the relative change from seawater controls: macrophyte-incubated seawater pH_e_ minus control seawater pH_e_, post-equilibration. If changes in seawater pH during incubation were due exclusively to decreases in CO_2_ and HCO_3_^-^ concentrations via macrophyte photosynthesis, then pH_e_ of macrophyte-incubated seawater should equal pH_e_ of control seawater after atmospheric re-equilibration, and pH_e_ shift would be 0. Alternatively, a difference in pH_e_ between macrophyte-incubated seawater and controls indicates macrophytes affected seawater chemistry other than through simple DIC depletion.

To test whether total alkalinity changed with macrophyte presence, TA was measured immediately after the 24-hour pH assay on 15 ml using an Alkalinity Titrator (Apollo SciTech Model AS-Alk2 SeaWATER gran titration) sample with 0.1 N HCl at 25°C calibrated with a TA standard (Batch 128, Andrew Dickson CO2QC Laboratory, Scripps Institute Oceanography). Specimens were randomized in water bath position and consequently also randomized in measurement order for TA, minimizing potential effects of machine drift. TA was measured as the concentration of titratable weak bases present relative to a reference pH at which DIC is 100% CO_2_. These analyses had a measurement uncertainty (s.d.) of ± 45 μmol/kg (1.9%) based on multiple assays of the standard. To make total alkalinity values comparable across assays from different seawater batches, TA was reported as change from seawater controls: macrophyte-incubated seawater TA minus control seawater TA, post incubation. If a TA shift is 0 μmol/kg, macrophytes did not change seawater at all during the incubation. Negative changes to TA indicate macrophyte-associated TA declines, while increased TA was indicated by positive values. To test whether pH*, pH_e_ or TA shifts were significantly different from seawater controls, we applied a two-tailed t-test by species.

In addition to determining how seaweeds change the pH of the water, we also determined the δ^13^C values of meristematic tissue. We analyzed ground tissue at the University of Chicago Stable Isotope facility. Detailed methods are reported elsewhere [13].

#### Evolution of CCMs using discrete CCM status and continuous pH*

We asked whether CCMs evolved differentially among families within the red and brown algae (Rhodophyta and Ochrophyta), by mapping pH* results and the incidence of CCMs to family-level phylogenies. We did not investigate evolutionary patterns in the Chlorophyta because of the limited number of green algae species prevalent in our flora. An ultrametric Bayesian phylogeny for Rhodophyta was adapted from [31] with a best-fit λ = 10. An ultrametric maximum likelihood phylogeny for Ochrophyta was digitized [32] and adapted from [33] with λ = 1000. Experimental pH* data were compiled from a review of 25 pH* studies [13], including this one (S3 and S4 Tables).

pH* was mapped as a continuous trait onto family-level trees using family averages. One hundred eleven Rhodophyta species were categorized into 30 families and subfamilies according to taxonomy in AlgaeBase [34] and 51 Ochrophyta species were similarly categorized into 18 families. We also examined CCMs as a discrete trait, by scoring CCM presence for species with mean pH* > 9.10 to account for potential artifacts in the assay [13].

CCMs have also been inferred from tissue δ^13^C values, where species lacking CCMs have lower δ^13^C values [9, 13,14] reflecting their use of CO_2_. Previous studies have found a strong negative relationship between pH* values and macrophyte tissue δ^13^C [9,13,14]. While a pH drift assay is an immediate measure of current CCM reliance and HCO_3_^-^ use over 24 hours, δ^13^C represents a time-integrated measurement of HCO_3_^-^ and CO_2_ incorporation into macrophyte tissue. Where pH* values were ambiguous in their CCM designation (8.90–9.10), we used δ^13^C tissue values to determine whether a CCM was present, using the cutoff value δ^13^C >-30‰ [9, 12]. Species with a pH* in this range that lacked corresponding isotope data (6 species) were excluded from the analysis to avoid false negative or positive results. For species in which pH* and carbon isotope values yielded strongly different CCM categorizations, we based our classification on isotope data. We therefore report both pH* data and C isotope values where available.

CCM presence was assigned to a family if at least one family member had a CCM. Evidence for CCMs from short-term pH* assay data largely correlated with time-integrated measurements of bicarbonate use and CCM presence using macrophyte tissue carbon stable isotopes, particularly in red algal species Only 3 Rhodophyte species were reassigned their CCM status based on incorporating isotope data, leading to a change in two Families to CCM presence (Peyssonneliaceae and Schizymeniaceae). Four Rhodophytes had pH* data between 8.90 and 9.10 without corresponding δ^13^C data and thus were excluded from the analysis. In the Ochrophyta, two species had pH* data between 8.90 and 9.10 without corresponding δ^13^C data and thus were excluded from the analysis. Seven Ochrophytes were reassigned CCM status based on incorporation of isotope data, leading to three families of Ochrophyta reassigned as having CCMs (Desmarestiaceae, Lessoniaceae and Scytosiphonaceae). Upon categorization of these three families as having CCMs, all 18 Ochrophyte families surveyed were designated as having CCMs, therefore only the continuous CCM trait pH* was tested for phylogenetic signal.

We performed ancestral state reconstruction and stochastic character mapping [35] on CCM presence/absence in the Phylum Rhodophyta using the R packages “ape” [36] and “phytools” [37] and an equal rates model as in [38]. Stochastic character mapping was performed 1000 times to obtain the average number of transitions between CCM presence and absence [39].

To test whether CCMs exhibited more or less divergence than expected if they were evolving under a null Brownian motion model of evolution, we calculated Blomberg's K for the continuous pH* trait [40] within Rhodophyta and Ochrophyta and Pagel's λ for the discrete CCM status trait [41] within only Rhodophyta. In a Brownian motion evolutionary model, the observed difference in traits between a pair of taxa is directly proportional to the estimated branch length since their lineages diverged. K ranges from 0 to infinity, where K = 0 indicates trait evolution is completely independent of phylogeny, K = 1 indicates Brownian motion evolution, K > 1 indicates that close relatives are more similar than expected, and K < 1 (the majority of values reported) indicates that close relatives are more divergent than expected [40]. We tested whether K was different from 1, indicating a departure from a Brownian motion model, by comparing our observed K values to a null distribution of K = 1 generated from our phylogenies using the R package “phytools”. For the discrete trait of CCM presence or absence by family, we calculated Pagel's λ using “phytools”. Pagel's λ varies from 0, where each phylogeny branch tip is independently derived (trait relationships are best represented by an unresolved 'star' phylogeny), to 1, where the internal, non-tip phylogenetic branches explain some of the shared ancestry of the traits and there is significant phylogenetic structure. To test for significance of λ, we used a likelihood ratio test to compare the calculated λ to λ = 1.

We tested for an effect of macroalgal Phylum membership (Chlorophyta, Rhodophyta, Phaeophyta) on seawater pH* and TA with an ANOVA, followed by a Kruskal-Wallis test if residuals were not normally distributed. To test whether families with CCMs had more species than families lacking CCMs, we obtained family richness data from AlgaeBase and applied a one-tailed t-test.

### Species-specific effects on the dissolved inorganic carbon (DIC) system

pH*, temperature, salinity and TA data were used to calculate DIC at 12°C as the sum of HCO_3_^-^, CO_3_^2-^ and CO_2_, as well as calcium and aragonite saturation states in final control and macrophyte-incubated seawater. We used the CO2sys_v2.1 Excel macro [42] under default constants [43], with pH scale set to NBS. We regressed pH* on TA to test whether they were related. To determine whether TA or pH* influenced the pH of equilibration with atmosphere (pH_e_), pH_e_ was regressed against TA and pH* in a multiple regression.

To compare how changes in TA and pH alter CO_2_, HCO_3_^-^ and CO_3_^2-^ availability, we compared observed proportions of these three DIC forms to expected proportions if TA remained constant. Using macrophyte-incubated seawater pH* and control seawater TA, we calculated the expected concentration of each DIC form over a range of pH* values. We then compared these expected values to the concentrations of CO_2_, HCO_3_^-^ and CO_3_^2-^ observed at the pH* and corresponding TA of macrophyte-incubated seawater (S1 Fig).

## Results

### pH* drift and Carbon Concentrating Mechanisms

Seawater nutrient concentrations varied among batches (S1 Table), however this did not strongly affect assay results of CCM presence or absence. Of 10 species assayed with multiple seawater batches, three had different pH* between batches (two-tailed t test, *C*. *pikeanum* t_5_ = 6.5, p = 0.001; *C*. *ruprechtiana* t_8_ = 5.9, p < 0.001; *A*. *coalita* t_8_ = 4.6, p = 0.002). Although pH* differed among trials in these three species, all trials consistently indicated pH* > 9.0 (*A*. *coalita*) or < 9.0 (*C*. *pikeanum*, *C*. *ruprechtiana*) within species. Some of the highest pH* values observed *(Ulva intestinalis*, 10.16 ± 0.06 standard error [s.e.]; *Urospora sp*., 10.23 ± 0.02 s.e.) were from assays in comparatively low-nutrient seawater (S1 Table), suggesting that seawater nutrients were not limiting to DIC depletion. Therefore, replicates from different seawater batches were pooled for species-level analyses.

**Fig 1:**
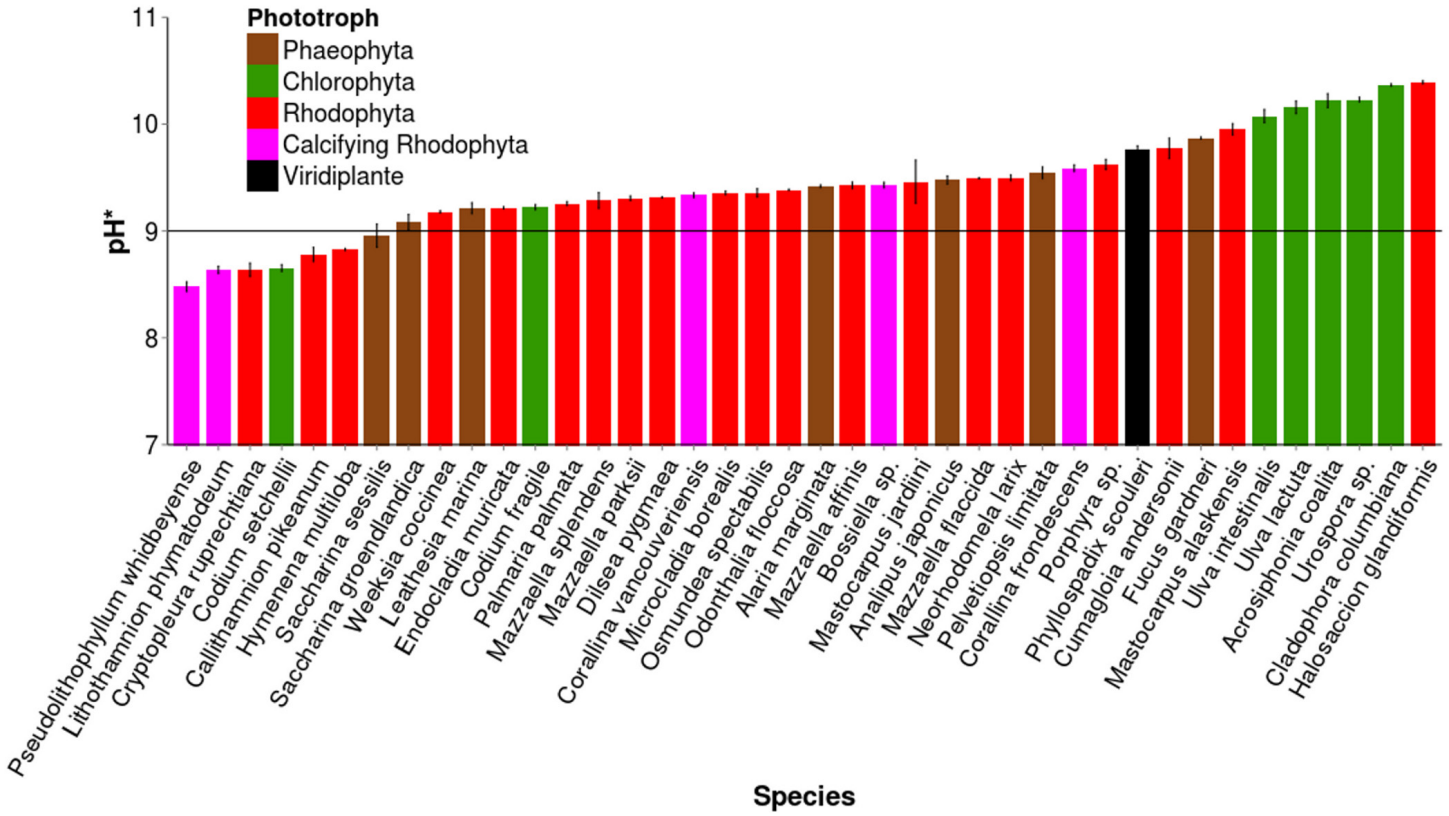
Mean pH* ± standard error (s.e.) for 39 species of intertidal seaweed and one surfgrass. Species with pH* > 9.0 (line) were designated as those that utilize HCO_3_^-^. Calcifying Rhodophyta species with pH* < 9.0 are crustose species, while calcifying Rhodophytes with pH* > 9.0 are articulated species.

pH* levels ranged from 8.48–10.39 (S2 Table). Thirty-two of 39 species increased seawater pH* > 9.0, indicating these species use HCO_3_^-^ and have active CCMs (Fig 1), consistent with other findings [4,7,25,26]. The angiosperm species *Phyllospadix scouleri* also had pH* indicative of a CCM. Species with CCMs showed differential efficiency in depleting dissolved inorganic carbon, based on a range of pH* from 9.08–10.39. Chlorophyta and Rhodophyta contained both species with and without active CCMs, while all Phaeophytes assayed had CCMs. pH* differed among Phyla (F_2, 36_ = 3.8, p = 0.032), with Chlorophyta driving pH* higher than Rhodophyta (Tukey HSD, p = 0.026). While seawater incubated with crustose calcifying species had pH* < 9.0, upright calcifying species increased seawater pH* > 9.0, indicating HCO_3_^-^ usage. Within-species pH* standard error ranged from 0.00–0.11 pH units (Fig 1, S2 Table), indicating that independent replicates of the same species produced consistent results and that the minority of partial individuals used in some replicates did not drive observed patterns. Species where partial individuals were used (*Alaria marginata*, *Saccharina groendlandica*, *S*. *sessile*, *Codium fragile*, *and Osmundea spectabilis*) were not outliers with respect to any seawater chemistry analysis, suggested that cutting the tissue had a minor effect on our results. *Mastocarpus jardiini* (± 0.20 pH units) was a notable exception to low within-species variation, but may comprise a multi-species complex [44].

After re-equilibration to atmospheric CO_2_ levels and thus initial seawater DIC values, the pH value at this equilibrium (pH_e_) of both control and treatment replicates was compared to initial seawater pH prior to incubation (time 0). Control pH_e_ did not differ from control initial pH (two-tailed paired t test, t_53_ = -1.0, p = 0.324). However, macrophyte-incubated seawater pH_e_ deviated from initial seawater controls by -0.35 ± 0.12 to +0.28 ± 0.03 s.e. pH units (S2 Table), but was unrelated to pH* (R^2^ = 0.102, F_1, 36_ = 4.1, p = 0.051). Values differed by Phylum (F_3, 35_ = 3.3, p = 0.032), with Chlorophyta having lower pH_e_ than Rhodophyta (Tukey HSD, p = 0.024). pH_e_ differences in macrophyte-incubated seawater compared to initial conditions suggest that macroalgae influence seawater chemistry in ways other than depleting DIC for photosynthesis.

### Evolutionary history of CCMs

Our evolutionary analysis of Ochrophyta: Phaeophyceae comprised species from 18 of 59 total families as defined by AlgaeBase [34] (31%). In Rhodophyta: Florideophyceae, we analyzed 29 of 95 total families (31%), excluding Bangiaceae (1 family). In both clades, species showed little variation in CCM status within-family (S3 and S4 Tables). In 13 of 20 Rhodophyta families in which there were data for multiple species, 100% of member species shared the same CCM designation (i.e. all species in the family had CCMs, or all lacked them; n = 2–10 species per family, S3 Table). This was also the case in 8 of 10 Ochrophyta families with more than one family member (n = 2–12 species per family, S4 Table). Ancestral state reconstruction indicated that CCMs are more likely to be ancestral in Rhodophyta (0.60 probability of CCM presence). Families with CCMs did not have higher species richness than families lacking CCMs in Rhodophyta (one-tailed t test, t_26,1_ = -1.22, p = 0.117). All families surveyed in Ochrophyta had species with CCMs, indicating that this trait is highly conserved within the brown algae.

**Fig 2:**
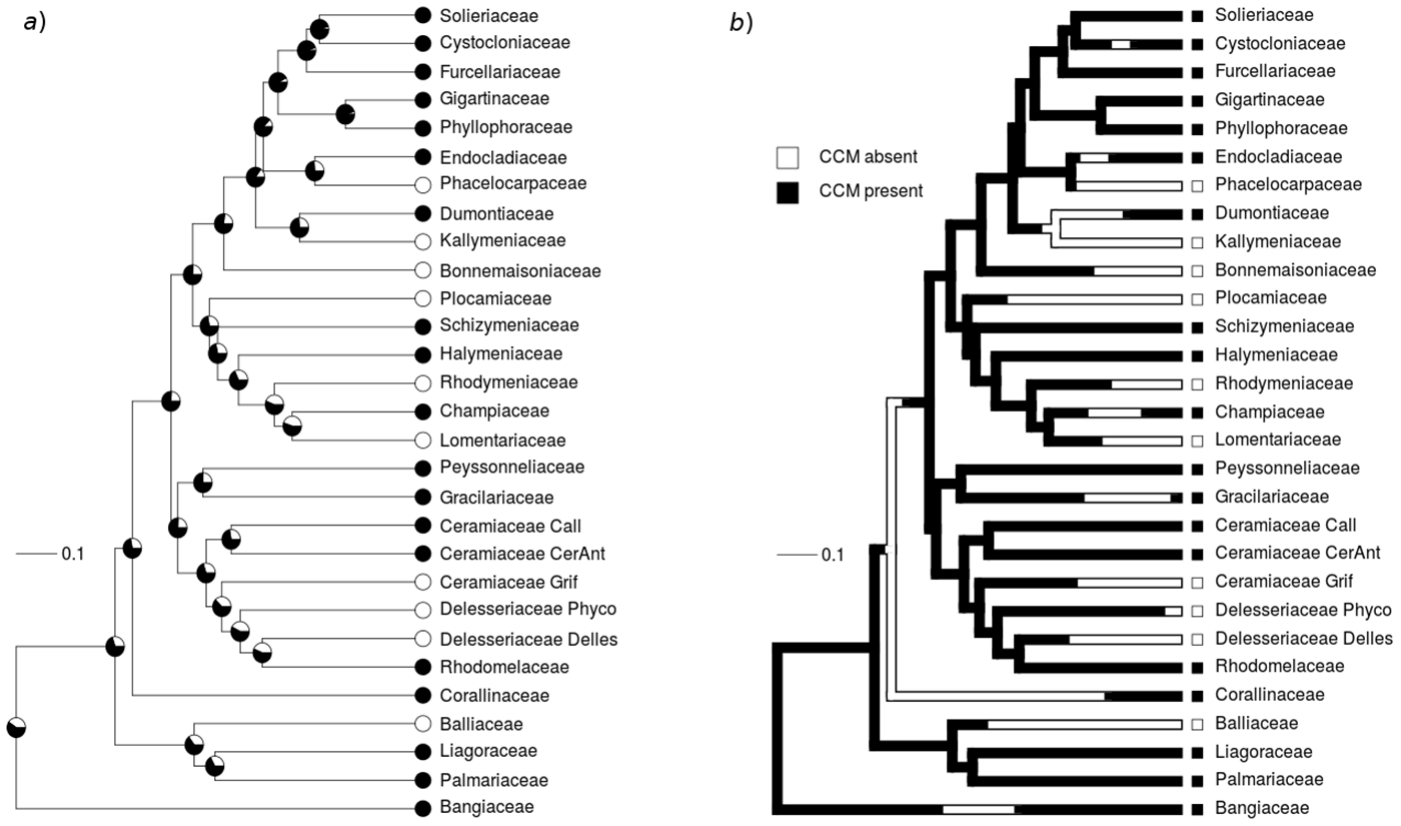
Ancestral state reconstruction and stochastic character mapping of Carbon Concentrating Mechanism (CCM) presence and absence in Rhodophyta. (A) Ancestral state reconstruction of CCM presence (black) and absence (white) in 30 families from Phylum Rhodophyta, in a phylogeny adapted from [31]. Pagel's λ = 4.10 x 10^−5^, p[λ = 1] = 0.010 and p[λ = 0] = 1.000, indicating no phylogenetic signal in the discrete trait of CCM presence or absence. (B) One of 1000 phylogenies generated depicting one possible trait reconstruction for CCM status in Rhodophyta. Trait reconstructions were generated through stochastic character mapping [37]. Changes may occur within branches because reconstructions depict not only the states at nodes but also the states at all points between nodes. Subfamilies are shown for Ceramiaceae and Delesseriaceae.

In contrast to the constant presence of CCMs in all Ochrophyta families surveyed, stochastic character mapping of CCM status in Rhodophyta revealed that CCMs have been lost and gained an equal number of times in the red algae (Fig 2*a* and 2*b*). In Rhodophyta, an average of 32.3 transitions (mode = 33) were observed between discrete CCM states in stochastic trait mapping (Fig 2*b*), with 16.2 transitions from CCM absence to presence (mode = 15), and 16.1 from CCM presence to absence (mode = 18).

Blomberg's K for Rhodophyta for the continuous pH* trait did not differ from K = 1 (K = 0.76, p = 0.280), suggesting that evolution of pH* or CCM efficiency in Rhodophyta exhibited significant phylogenetic signal consistent with the expectations of a Brownian motion model. Similarly, in Ochrophyta, Blomberg's K for pH* also did not differ from K = 1 (K = 0.82, p = 0.627). However, Pagel's λ for the discrete trait CCM presence in Rhodophyta differed from 1 (λ = 4.10 x 10^−5^, p = 0.010), and was comparable to λ = 0 (p = 1.000), i.e., a star phylogeny, again indicating that families' discrete CCM status are largely independently derived in red algal families.

### Species-specific effects on seawater chemistry

**Fig 3:**
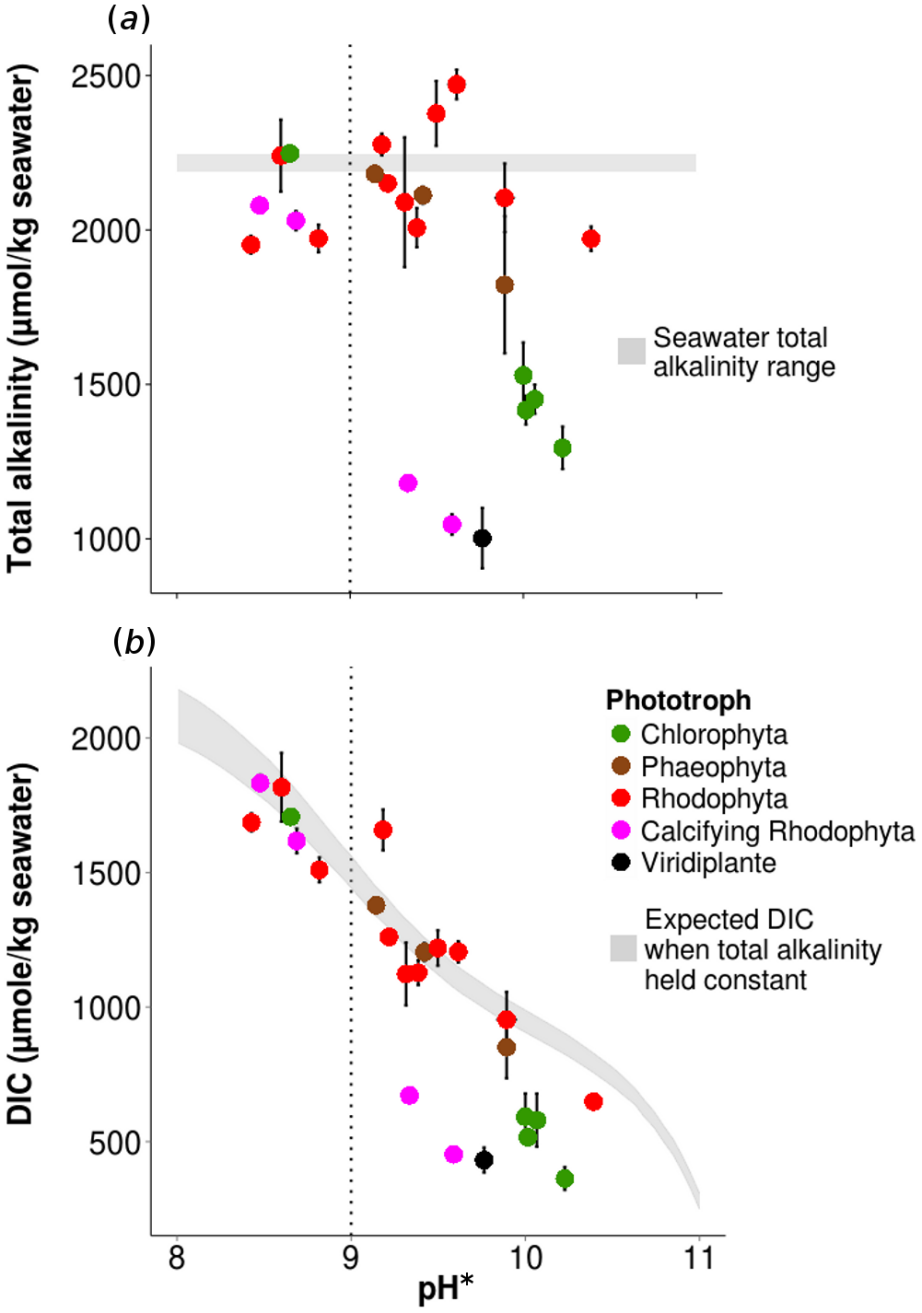
Seawater total alkalinity and DIC concentrations after 24 hour incubation under light with 23 seaweed species and one surfgrass species (2–6 reps). (A) Mean seawater TA ± standard error (s.e.) versus pH*. Gray envelope indicates control seawater TA. Dotted line indicates break point for HCO_3_^-^ use (pH > 9.0). (B) Mean calculated DIC ± s.e. versus pH*. Gray envelope indicates expected DIC-pH relationship if TA is held constant at ambient seawater levels.

Although pH* indicated 2 groups of species, where a CCM was either indicated or not, seawater alkalinity patterns differed among species in unexpected ways. Shifts in TA differed among species as pH increased: at high pH, some species depleted TA while others increased it (Fig 3*a*). TA shifts ranged from depletions of up to 1170 μmol/kg seawater, to increases of up to 255 μmol/kg seawater and differed significantly from controls in 16 of 24 species evaluated (Fig 3*a*, S2 Table); only *Porphyra sp*. increased TA. Although four of five green algal species decreased TA more than any other fleshy macroalgal species (Fig 3*a*), TA did not vary by Phylum (Kruskal-Wallis χ^2^ = 3.09, df = 2, p = 0.213). Initial seawater TA on a collected batch was 2192 μmol/kg seawater, compared to 2198 μmol/kg post-incubation for that batch, indicating that seawater TA in controls changed little during incubation. Calcium and aragonite saturation also varied among species (S2 Table).

Deviation in seawater pH_e_ from initial conditions was positively related to TA, but not to pH* (S2 Fig; multiple regression, R^2^ = 0.466, F_1,21_ = 8.3, p = 0.012, and p = 0.168, respectively), supporting the hypothesis that changes in water chemistry by macrophytes via change in TA, rather than incomplete re-equilibration, caused shifts in pH_e_. TA was negatively related to pH* (R^2^ = 0.203, F_1,21_ = 5.4, p = 0.031, Fig 3*a*).

Thirteen species depleted significantly more DIC than expected if TA had remained constant, while only *Porphyra sp*. increased DIC (149 ± 28 s.e. μmol/kg seawater, t_6_ = 2.8, p = 0.029, Fig 3*b*, S3 Table). Deviation from expected DIC was greater for articulated calcifying Rhodophytes than crustose species (-711 ± 30 μmol/kg seawater versus -139 ± 23 μmol/kg seawater, respectively). Chlorophytes also showed strong depletion in DIC up to -524 ± 38 μmol/kg seawater, and the one angiosperm species surveyed showed the greatest observed depletion of DIC relative to the expected DIC value (*P*. *scouleri*, -757 ± 72 μmol/kg seawater).

**Fig 4:**
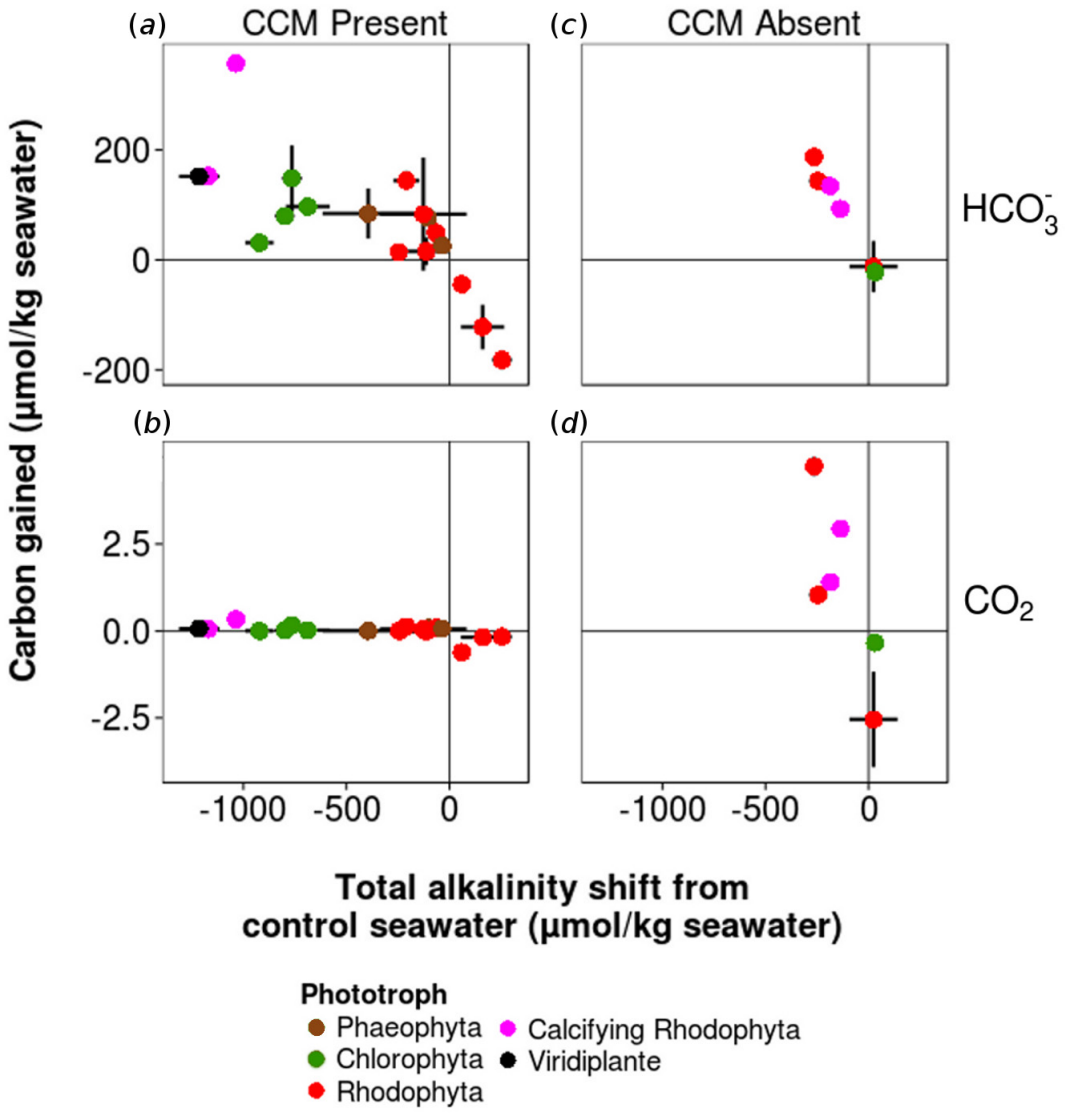
Carbon gained through TA shifts. (A) Mean HCO_3_^-^ ± standard error (s.e.) gained versus mean TA shift ± s.e. in seawater incubated with CCM-present macrophytes. Mean HCO_3_^-^ ± standard error (s.e.) gained versus mean TA shift ± s.e. in seawater incubated with CCM-present macrophytes. (B) CO_2_ gained versus TA shift in seawater incubated with CCM-present macrophytes. (C) HCO_3_^-^ gained versus TA shift in seawater incubated with CCM-absent macrophytes. (D) CO_2_ gained versus TA shift in seawater incubated with CCM-absent macrophytes. Solid lines indicate control seawater axes for all panels.

Macrophyte-driven changes in TA can change HCO_3_^-^, CO_2_ and CO_3_^2-^ concentrations relative to the concentrations if TA was unchanged. Through decreasing TA and thus decreasing pH, 13 species significantly increased HCO_3_^-^ concentrations and 12 species significantly increased CO_2_ concentrations (Fig 4, S5 Table) at the expense of CO_3_^2-^. Increases in HCO_3_^-^ ranged from 14.29 ± 0.85 to 357.59 ± 7.59 s.e. more μmol/kg seawater, corresponding to 144–1571% of expected values (Fig 4*a*). Increases in CO_2_ ranged from 0.09 ± 0.01 to 4.73 ± 0.28 μmol/kg, reflecting proportionately large changes (141–83000%) owing to its negligible concentration at higher pH. The single species that increased TA (*Porphyra sp*.) led to a CO_2_ loss of 0.17 ± 0.02 μmol/kg seawater, 40% of expected values under constant TA (Fig 4*b*, S5 Table). Because [CO_3_^2-^] is inversely proportional to [CO_2_] + [HCO_3_^-^] (S1 Fig), decreases in CO_3_^2-^ approximately mirrored increases in HCO_3_^-^ (S3 Fig).

The majority of macrophytes with CCMs (pH* > 9.0) that shifted seawater TA showed small but significant changes in CO_2_ (Fig 4*b*). Because HCO_3_^-^ users raised seawater pH* well above 9.0 —after which [CO_2_] < 1 μmol/kg seawater—TA-induced changes to [CO_2_] were largely negligible (S1 Fig). Six species did not appear to access bicarbonate based on their pH* value, however they significantly decreased total alkalinity (S1 Table). For these species, TA-induced decreases in pH* quickly fall within a pH range with non-negligible CO_2_ concentrations (S1 Fig), causing increases in [HCO_3_^-^] and driving [CO_2_] to the highest levels observed in the study (Fig 4*c* and 4*d*).

## Discussion

### CCM evolution in Rhodophyta and Ochrophyta

Our analysis of 31% of families in each of Rhodophyta and Ochrophyta showed consistent patterns of CCM evolution between red and brown algal lineages, but differing patterns between categorization of CCMs as a continuous versus discrete trait. Evolution of the continuous trait pH*, to an extent a measure of CCM efficiency, exhibited a phylogenetic signal similar to a Brownian motion model of evolution in Rhodophyta and Ochrophyta, where phenotypic differences between taxa are directly proportional to the branch lengths characterizing their independent evolutionary history (Fig 2a). However, when classifying CCMs as a discrete presence or absence trait, CCMs are highly labile in Rhodophyta with multiple losses and gains inferred, but are present in every Ochrophyta family surveyed and thus highly conserved. The discrete trait of CCM presence in Rhodophyta was not significantly different from a star phylogeny, indicating that phylogenetic signal in CCM presence was weak and suggesting that different lineages experienced different rates of CCM evolution in response to their environment [40].

While we inferred many losses of CCMs in the red algal lineages investigated, loss of CCMs is rare in terrestrial plant lineages. CCMs have evolved as C_4_ photosynthesis more than 62 times independently in flowering plants and are considered highly convergent in flowering plants [45]. In the grass family Poaceae alone (100 million years old [46]) there are 22–24 inferred origins of C_4_ photosynthesis, and 1 potential loss [47], in contrast to the 35 transitions observed in Rhodophyta. The high number of CCM losses in Rhodophyta (17) mirrors the number of transitions observed in some behavioral traits of birds in the literature [39]. However, strict comparisons of transitions in Rhodophyta with other studies could not be made because clade age and size varied greatly among studies.

Loss and gain of CCMs in Rhodophytes indicate that selection for CCMs may depend upon environmental parameters, including local and global habitat features, and source inorganic carbon availability. The ability of marine algae to utilize different sources of inorganic carbon provides interesting contrasts with terrestrial plants and may contribute to biogeographic patterns. In the poles, where temperatures are low, CO_2_ is more easily absorbed from the atmosphere into the oceans, and in the low-light subtidal zone, where macrophytes are constantly submerged compared to the intertidal zone, CCMs are less common [13,14]. This environment would appear not to select for CCMs, however the Ochrophyta: Phaeophyceae is one of the few lineages that largely originated and diversified in temperate latitudes [48], is proportionately more prevalent at lower tidal levels, and comprises species with CCMs in every Ochrophyta family investigated in this study. However, this group does show less phylogenetic structure than expected when modeling CCM efficiency as a continuous pH* value. Clade age may also contribute to the large number of transitions and low phylogenetic signal observed in the Florideophyceae red algae, a 500–600 million year old clade [49], compared to 150–200 myo Phaeophyceae [33].

CCMs in marine macrophytes have demonstrated plasticity in functionality by depth [14], and pyrenoids, chloroplast components of CCMs in some taxa, have been lost and gained multiple times in Chlorophyta [5]. In contrast, CCM evolution in terrestrial plants is strongly linked to the proportion of vascular bundle sheath tissue [50]. As a complex physiological trait without assignment to a single morphological structure, it is difficult to determine the mechanism by which a marine lineage loses or gains a CCM. Although we inferred the presence of a CCM here through the functional assay of pH drift and previously through inferences from tissue δ^13^C [13], CCMs can also be identified by searching for genes suspected of CCM activity (e.g. [51]), although this is limited to genes with known sequences and function. As DNA sequence variation, gene presence, and carbon utilization become better understood, our knowledge of CCM genealogy will continue to develop. Because CCMs can be costly to utilize [6], local and long-term patterns in carbon supply may change their selective advantage.

### Macrophytes exert species-specific effects on seawater chemistry

Most macroalgal species tested increased pH > 9.0, though their effects on seawater TA varied greatly (Figs 1 and 3). Overall, 92% of species had either a CCM or the ability to shift TA, with Chlorophytes having significantly higher pH* than Rhodophytes; excluding *C*. *setchellii*, Chlorophytes assayed here are characterized as fast-growing, early successional species [52] that may benefit from bicarbonate access. Of 6 possible combinations of CCMs and TA shifts—CCM presence or absence with TA increase, decrease, or no effect– 5 were observed. We never observed species that lacked CCMs increase TA, and few species lacking CCMs decreased TA. Several species of red (*E*. *muricata*, *D*. *pygmaea*, *M*. *alaskensis*) and brown seaweeds (*A*. *marginata*) with CCMs had no effect on seawater TA. The only species with a CCM that increased TA (*Porphyra sp*.) also decreased CO_2_ (t_3_ = -3.6, p = 0.038), however the change was small (-0.17 μmol/kg seawater CO_2_). Among the remaining CCM-TA trait combinations, we focus on the groups that decreased TA and thus increased DIC: 1) species with CCMs that decreased TA (the majority of species surveyed), and 2) species lacking CCMs that decreased TA.

Species with CCMs that decreased TA were represented across all three algal phyla and the angiosperm assayed. TA decreases likely resulted from macrophyte-facilitated proton addition to seawater. Biological uptake and transformation of cations such as positively-charged ammonium generates H^+^ and decreases TA, while uptake of anions including negatively-charged nitrate and sulfate generates OH-, which increases TA [53]. In previous studies, the brown alga *Ascophyllum* decreased TA through cation generation [18]. However, these effects are relatively small and do not fully explain the large TA declines we observed (on the order of hundreds of units).

Calcifying algae interact with the ocean carbon system to form a CaCO_3_ skeleton, which may further alter local seawater chemistry. In the proposed ‘trans’ model of calcification, active transport of Ca^2+^ and passive diffusion of CO_2_ forms CaCO_3_ and releases H+ ions extracellularly [54]. Whether this proton extrusion decreases pH locally more than the pH increase from photosynthesis [55], and further increases CO_2_ for photosynthesis, remains to be determined. In calcifying freshwater green algae, Ca^2+^ and HCO_3_^-^ uptake leads to precipitation of CaCO_3_ and production of CO_2_ [6]. In our study, articulated corallines (*C*. *frondescens*, *C*. *vancouveriensis*) increased pH* > 9.0 and strongly decreased TA. Crustose corallines (*P*. *whidbeyense* and *L*. *phymatodeum*), in contrast, had little effect on either pH or TA, perhaps because their comparatively slower metabolism [56] prevented detection of the influence of photosynthesis or calcification on seawater chemistry over 24 hours. Calcium carbonate skeletons in macroalgae are usually assumed to function as a defense against enemies [57]. Our results support an alternative hypothesis: that macroalgal calcium carbonate skeletons are a byproduct of manipulating TA to facilitate photosynthesis in environments where DIC may be depleted, a mechanism proposed for calcification in coccolithophores [58] and macrophytes [54].

Species lacking CCMs that decreased TA (such as *C*. *ruprechtiana*, *H*. *multiloba*) likely released or took up other ionic components independent of the carbonate system. Decreased TA benefits these CO_2_-dependent species because CO_2_ becomes relatively more available (Fig 3*d*). An intriguing consideration is that TA reduction may be a mechanism beyond active carbon transport to locally increase CO_2_ access. For species that cannot otherwise access HCO_3_^-^; TA reductions increased CO_2_ up to 4 μmol/kg seawater. For those that have a recognized CCM, a reduction in TA may present another means for concentrating carbon and thus may too be considered a CCM.

Just as some CCMs create external 'acid zones' in boundary layers to convert HCO_3_^-^ to CO_2_ by pumping H+ into the boundary layer [59], decreased TA may create local 'low TA zones' that generate lower pH regions to adjust the buffering capacity of the water. Organic bases can have significant impacts on seawater TA in cultures of marine macroalgae and in marine sites characterized by high biological activity and restricted water mixing [60]. Regardless of the mechanism of a decrease in TA, there is growing evidence that photosynthetic carbon uptake extends beyond the dynamics of CO_2_ [22]. The net result of a reduction in alkalinity is increased availability of DIC, both as HCO_3_^-^ and CO_2_, for photosynthesis (Fig 4), and carbon capture via photosynthesis may be higher than expected on the basis of CO_2_ dynamics alone. That both the angiosperm *P*. *scouleri* and calcifying algae had large TA decreases but no significant change in pH_e_ from controls may point to the diversity of mechanisms by which macrophytes—angiosperms, calcifying and non-calcifying macroalgae—interact with seawater. Carbon acquisition thus likely has multiple underlying mechanisms that deserve further study.

We investigated whether alga shifts in TA, by altering the point at which [CO_2_] becomes negligible, could have affected the cutoff for a CCM and caused the mis-assignment of CCM presence/absence. Specifically, species that reduce TA (leading to lower pH) and have pH* < 9 might still have CCM and species that increase TA (leading to higher pH) and have pH* > 9 might not have a CCM. We calculated whether this shift may have affected our results by using CO2calc to calculate pCO_2_ in control seawater at pH = 9 under measured average nutrient, salinity, temperature, and TA values, and then calculated what the measured pH should be at that pCO_2_ level under altered TA conditions for each of the 5 algal species meeting the above criteria. The adjusted pH cutoff ranged from 8.965 to 9.030, depending on species. As all pH* were ≤ 8.84 for the four non-CCM/TA reducer species, and 9.62 for the CCM/TA increasing species (S2 Table), in no case would the CCM classification have changed.

Although macrophytes were rinsed and assayed in sterilized seawater, macrophytes likely hosted microbial populations that may have affected pH* and TA. To generate the range of observed pH* values and TA shifts however, microbial communities would need to be remarkably distinct among macroalgal species to cause shifts ranging from 0–2.4 pH units, or decreases in TA of up to half that of control seawater. Ascribing the range of pH* and TA shifts observed among species to the relatively high algal biomass, rather than microbial communities, seems more parsimonious. Dissolved organic carbon, though possibly an exudate in the algae in our experiments, also is unlikely to differ by amounts that would drive such large changes in TA. Further, there is strong concordance among the assayed species between phototroph tissue δ^13^C —a strong indicator of CCM status—and pH assay results [13].

Calcite and aragonite saturation states were higher in seawater incubated with macrophytes (S1 Table) and may have led to some spontaneous CaCO_3_ precipitation at high pH*, which could drastically lower total alkalinity. However, if a purely abiotic pH-dependent reaction was behind some of the large TA drops observed, it would not lead to consistent differences among species with similar pH* across multiple independent replicates (Fig 3*a*). If observed TA reductions were generated by spontaneous external calcification, then the consistent species-specific responses imply that some macroalgae possess traits that promote the process, such as morphologies or chemical exudates that promote CaCO_3_ enucleation, whereas others do not. Such external calcium carbonate precipitation would be distinct from carbon concentrating mechanisms, an interesting finding worth further investigation. Overall, both total alkalinity shifts and pH* values were consistent within species among intra-study replicates, inter-study replicates, and seawater batches, indicating that findings were due to macrophyte species identity rather than methodological artifacts.

### Potential implications of CCMs for macrophyte communities

While macrophyte-induced shifts in TA may be non-linear, not occurring until the surrounding seawater approaches the species' pH* [18], the size of the diffusion boundary layer and effects of water mixing may create conditions in nearshore environments where these mechanisms become locally important in carbon cycling. In our study, 4 g of macrophyte shifted TA in 125 mL of water (32 g/L). Comparable studies with biomass-to-seawater proportions as low as 0.007 g/mL in 700 mL chambers report similar TA shifts [18]. The boundary layer can be very small in communities dominated by crust-forming species, but may be much thicker in more structurally diverse communities [11], indicating that observed TA effects could be far-reaching in areas of low water motion. Furthermore, although the experiments are small-scale, the algal biomass to volume ratio used is actually very similar to those calculated at a typical high tide (the most dilute conditions) from field data (3–30 g/L; [61]).

While current techniques cannot probe TA changes at boundary layer scales, pH shifts of more than 2 units have been observed in boundary layers of algal species and biofilms under realistic flow and pH regimes [62,63]. Macrophyte-driven elevated pH at larger scales along shoreline waters indicates species may compete for DIC [1,64]. CCMs may allow macroalgae to be more productive in slow-moving seawater, where rates of CO_2_ supply can be reduced [10]. Excluding *C*. *setchellii*, Chlorophytes assayed here are characterized as fast-growing, early successional species [52] that may benefit from bicarbonate access. Chlorophytes such as species in the cosmopolitan genus *Ulva* can dominate areas of periodic low flow including tidepools [64], and embayments where it blooms in high densities [65,66]. Macrophytes with CCMs have extremely variable community abundances, from 0–100% coverage in communities surveyed thus far [7,14]. The great interspecies differences in carbon uptake demonstrated in our study and others suggests that carbon use and its local seawater effects may be important to intra-and interspecific interactions in marine macrophyte communities. Thus, in environments where algae experience periods of competition for carbon, CCMs may be a trait important to colonization and persistence.

## Supporting Information

S1 Fig Dissolved inorganic carbon proportions relative to pH. A) Log of [HCO_3_^-^], [CO_3_^2-^] and [CO_2_] as a function of pH. Grey line indicates concentrations at control seawater pH. While pH is an emergent property of dissolved inorganic carbon concentrations (B), it is often depicted on the x axis, as these values are somewhat interdependent.
(PDF)

S2 Fig Mean change ± SEM in observed versus expected pH_e_ vs total alkalinity in 39 species of seaweed and 1 surfgrass. All units in μmol/kg seawater. Filled circles depict species with CCMs present, open circles CCMs are absent. N = 111.
(PDF)

S3 Fig Mean change ± SEM in observed versus expected [CO32-] vs total alkalinity shift in μmol/kg seawater. (A) 17 species of seaweed and one species of surfgrass with CCMs, and (B) 6 species of seaweed without CCMs. N = 111.
(PDF)

S1 Table Source pH, temperature, salinity and nutrient profile of seawater collected for pH* assays. All concentrations are in μM.
(PDF)

S2 Table pH* and ΔpHe ± standard error (s.e.) for 39 species of intertidal seaweed and 1 species of surfgrass. Total alkalinity (TA) ± s.e. for 24 species. ΔpHe is calculated as observed pHe of macrophyte-incubated seawater minus pHe of seawater with seaweed absent, post incubation and requilibrium. † indicates calcifying species. ‡ indicates crust-forming calcifying species. N indicates number of individuals per species. p is p-value for a one-tailed *t*-test for shift in total alkalinity. Bolding indicates p-value less than 0.050. Ω Ar and Ω Ca indicates the saturation state of aragonite and calcium, repsectively. *Corallina frondescens* has only 1 replicate for pHe. CCM present indicates pH* > 9.0 if 'yes' and pH* < 9.0 if 'no.' TA shifts are reported as the TA of macrophyte-incubated seawater minus the TA of control seawater, post incubation. If a TA shift is 0, macrophytes did not change seawater at all during the incubation. TA shifts indicate whether changes in total alkalinity were significantly higher than 0 ('increase' relative to control seawater), lower than 0 ('decrease' relative to control seawater) or not different from 0 (not significant, 'n.s.', no effect of macrophyte incubation). Where multiple seawater batches are listed, this indicates species replicates were run across multiple trials, where a some specimens were run with batch x and others with batch y.
(PDF)

S3 Table Mean pH* for each of 111 species from Phylum Rhodophyta. Species data are compiled and averaged by family membership into 30 families for ancestral state reconstruction and stochastic character mapping. Mean species pH* are taken from a meta-analysis of 25 pH* studies (Stepien 2015). † indicates data from this study contributed to the species mean. Families in which at least one member has a CCM were designated as having CCMs. Grey shading indicates families and species that are categorized as having CCMs. The cutoff for CCM presence in individual species was pH* > 9.05.
(PDF)

S4 Table Mean pH* for each of 51 species from Phylum Ochrophyta. Species data are compiled and averaged by family membership into 18 families for ancestral state reconstruction and stochastic character mapping. Mean species pH* are taken from a meta analysis of 25 pH* studies (Stepien 2015). † indicates data from this study contributed to the species mean. Families in which at least one member has a CCM were designated as having CCMs. Grey shading indicates families and species that are categorized as having CCMs. The cutoff for CCM presence in individual species was pH* > 9.05.
(PDF)

S5 Table Changes in seawater carbon concentrations after incubation with 23 species of macroalgae and 1 species of surfgrass. ΔDIC is the deviation of Dissolved Inorganic Carbon concentration from expected DIC depletion under control Total Alkalinity conditions. ΔHCO_3_^-^ and ΔCO_2_ are carbon gained due to TA-induced pH shifts driven by macroalgae. N indicates number of individuals per species. p-value is for a two-tailed *t*-test for changes in carbon concentrations. † indicates articulated calcifying species. ‡ indicates crust-forming calcifying species. Bolding indicates p-value less than 0.050.
(PDF)
