# Supplementary material for: Functional Traits for Carbon Access in Macrophytes
Source: PLoS One. 2016 Jul 14;11(7):e0159062. doi: 10.1371/journal.pone.0159062 (PMC4944969; doi:10.1371/journal.pone.0159062)
Supplement: S1 Fig — A) Log of [HCO3-], [CO32-] and [CO2] as a function of pH. Grey line indicates concentrations at control seawater pH. While pH is an emergent property of dissolved inorganic carbon concentrations (B), it is often depicted on the x axis, as these values are somewhat interdependent. (PDF) [file pone.0159062.s001.pdf]

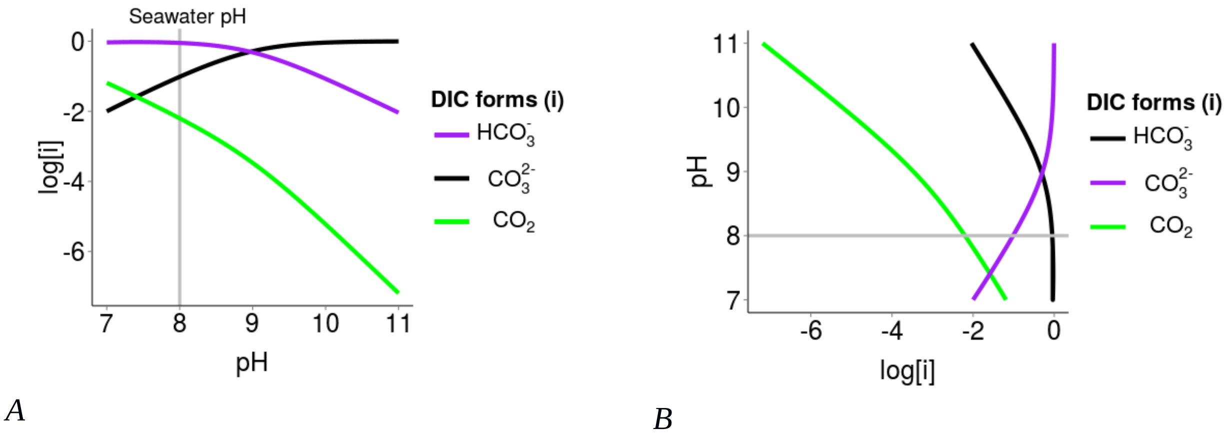

S1 Figure. **Dissolved inorganic carbon proportions relative to pH.** *A*) Log of [HCO_3^-], [CO_3^{2-}] and [CO_2] as a function of pH. Grey line indicates concentrations at control seawater pH. While pH is an emergent property of dissolved inorganic carbon concentrations (*B*), it is often depicted on the x axis, as these values are somewhat interdependent.
