# Supplementary material for: Functional Traits for Carbon Access in Macrophytes
Source: PLoS One. 2016 Jul 14;11(7):e0159062. doi: 10.1371/journal.pone.0159062 (PMC4944969; doi:10.1371/journal.pone.0159062)
Supplement: S2 Fig — All units in μmol/kg seawater. Filled circles depict species with CCMs present, open circles CCMs are absent. N = 111. (PDF) [file pone.0159062.s002.pdf]

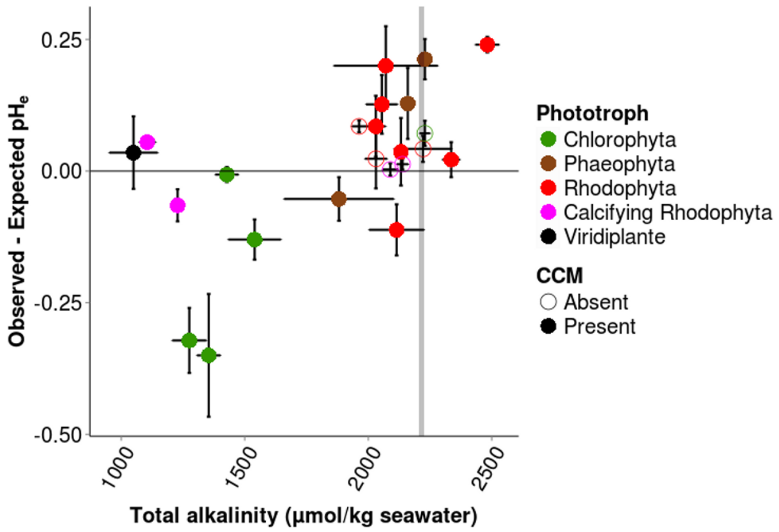


**S2 Figure. Mean change ± SEM in observed versus expected $pH_e$ vs total alkalinity in 39 species of seaweed and 1 surfgrass.** All units in µmol/kg seawater. Filled circles depict species with CCMs present, open circles CCMs are absent. N = 111.
