# Supplementary material for: Functional Traits for Carbon Access in Macrophytes
Source: PLoS One. 2016 Jul 14;11(7):e0159062. doi: 10.1371/journal.pone.0159062 (PMC4944969; doi:10.1371/journal.pone.0159062)
Supplement: S3 Fig — (A) 17 species of seaweed and one species of surfgrass with CCMs, and (B) 6 species of seaweed without CCMs. N = 111. (PDF) [file pone.0159062.s003.pdf]

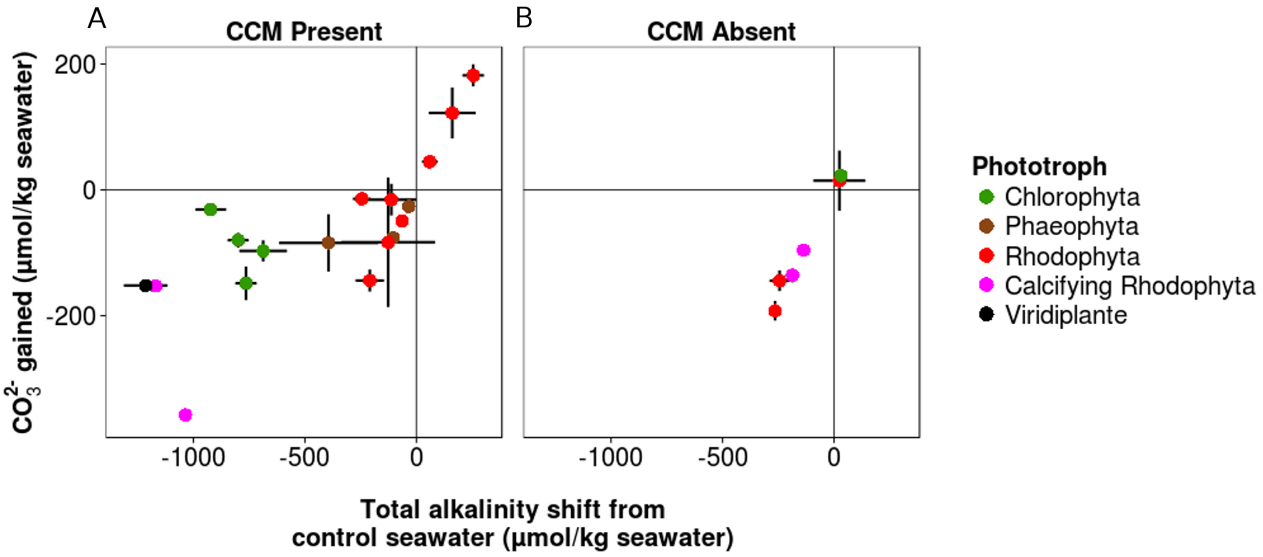

S3 Figure. **Mean change ± SEM in observed versus expected [CO_3^{2-}] vs total alkalinity shift in μmol/kg seawater.** (*A*) 17 species of seaweed and one species of surfgrass with CCMs, and (*B*) 6 species of seaweed without CCMs. N = 111.
