# Supplementary material for: Functional Traits for Carbon Access in Macrophytes
Source: PLoS One. 2016 Jul 14;11(7):e0159062. doi: 10.1371/journal.pone.0159062 (PMC4944969; doi:10.1371/journal.pone.0159062)
Supplement: S1 Table — All concentrations are in μM. (PDF) [file pone.0159062.s004.pdf]

**S1 Table. Source pH, temperature, salinity and nutrient profile of seawater collected for pH* assays.** All concentrations are in μM.

| seawater batch | date collected | source pH | source temp (ºC) | source salinity | [$PO_4$] | [$Si(OH)_4$] | [$NO_3$] | [$NO_2$] | [$NH_4$] |
|---|---|---|---|---|---|---|---|---|---|
| SW04 | 06/19/13 | 8.11 | 12.2 | 35 | - | - | - | - | - |
| SW05 | 06/21/13 | 7.83 | 12.7 | 34 | - | - | - | - | - |
| SW06 | 06/21/13 | 7.83 | 12.7 | 34 | - | - | - | - | - |
| SW07 | 06/24/13 | 7.97 | 12.7 | 34 | - | - | - | - | - |
| SW08 | 06/25/13 | 8.03 | 13.2 | 32 | 1.85 | 46.93 | 20.67 | 0.27 | 0.82 |
| SW09 | 06/29/13 | 8.34 | 18.8 | 30 | - | - | - | - | - |
| SW10 | 06/30/13 | 8.33 | 19.4 | 29 | 0.17 | 8.23 | 0.14 | 0.03 | 0.09 |
| SW11 | 07/06/13 | 8.24 | 14.0 | 32 | 0.65 | 22.08 | 3.49 | 0.18 | 0.09 |
| SW12 | 07/08/13 | 8.08 | 13.2 | 32 | - | - | - | - | - |
| SW13 | 07/09/13 | 8.00 | 14.4 | 33 | 1.73 | 48.89 | 15.44 | 1.06 | 0.62 |
| SW14 | 07/13/13 | 7.91 | 14.5 | 32 | 1.78 | 44.95 | 20.10 | 0.35 | 0.76 |
