# Supplementary material for: Functional Traits for Carbon Access in Macrophytes
Source: PLoS One. 2016 Jul 14;11(7):e0159062. doi: 10.1371/journal.pone.0159062 (PMC4944969; doi:10.1371/journal.pone.0159062)
Supplement: S2 Table — Total alkalinity (TA) ± s.e. for 24 species. ΔpHe is calculated as observed pHe of macrophyte-incubated seawater minus pHe of seawater with seaweed absent, post incubation and requilibrium. † indicates calcifying species. ‡ indicates crust-forming calcifying species. N indicates number of individuals per species. p is p-value for a one-tailed t-test for shift in total alkalinity. Bolding indicates p-value less than 0.050. Ω Ar and Ω Ca indicates the saturation state of aragonite and calcium, repsectively. Corallina frondescens has only 1 replicate for pHe. CCM present indicates pH* > 9.0 if 'yes' and pH* < 9.0 if 'no.' TA shifts are reported as the TA of macrophyte-incubated seawater minus the TA of control seawater, post incubation. If a TA shift is 0, macrophytes did not change seawater at all during the incubation. TA shifts indicate whether changes in total alkalinity were significantly higher than 0 ('increase' relative to control seawater), lower than 0 ('decrease' relative to control seawater) or not different from 0 (not significant, 'n.s.', no effect of macrophyte incubation). Where multiple seawater batches are listed, this indicates species replicates were run across multiple trials, where a some specimens were run with batch x and others with batch y. (PDF) [file pone.0159062.s005.pdf]

SUPPORTING INFORMATION

S2 Table. **pH*, ΔpHe ± standard error (s.e.), Total Alkalinity and tissue $\delta^{13}C$ for 39 species of intertidal seaweed and 1 species of surfgrass**. Total alkalinity (TA) ± s.e. for 24 species. ΔpHe is calculated as observed pHe of macrophyte-incubated seawater minus pHe of seawater with seaweed absent, post incubation and requilibrium. † indicates calcifying species. ‡ indicates crust-forming calcifying species. N indicates number of individuals per species. p is p value for a one-tailed t test for shift in total alkalinity. Bolding indicates p value less than 0.050. Ω Ar and Ω Ca indicates the saturation state of aragonite and calcium, repsectively. *Corallina frondescens* has only 1 replicate for pHe. CCM present indicates pH* > 9.0 if 'yes' and pH* < 9.0 if 'no.' TA shifts are reported as the TA of macrophyte-incubated seawater minus the TA of control seawater, post incubation. If a TA shift is 0, macrophytes did not change seawater at all during the incubation. TA shifts indicate whether changes in total alkalinity were significantly higher than 0 ('increase' relative to control seawater), lower than 0 ('decrease' relative to control seawater) or not different from 0 (not significant, 'n.s.', no effect of macrophyte incubation). Where multiple seawater batches are listed, this indicates species replicates were run across multiple trials, where some specimens were run with batch x and others with batch y. $\delta^{13}C$ values are sourced from [13].

Division	Taxa	CCM present	TA shift	pH* (s.e)	N	$\delta^{13}C$ (s.e.)	N	ΔpH_e (s.e.)	p ΔpH_e	TA (s.e.) µmol/kg SW	N	ΔTA (s.e) µmol/kg SW	p ΔTA	Ω Ca (s.e.)	Ω Ar (s.e.)	Ω Ca (s.e.) control	Ω Ar (s.e.) control	Seawater batch
Chlorophyta	*Acrosiphonia coalita*	yes	decrease	10.22 (0.07)	10	-17.55	1	-0.01 (0.01)	0.662	1427.75 (46.66)	4	-799.50 (53.50)	**< 0.001**	10.20 (0.46)	6.47 (0.29)	3.03 (0.19)	1.92 (0.12)	4, 14
	Cladophora columbiana	yes	-	10.37 (0.01)	6	-13.77	1	-0.02 (0.05)	0.700	-	-	-	-	-	-	-	-	8
	Codium fragile	yes	-	9.22 (0.02)	6	-14.27 (1.02)	14	0.19 (0.09)	0.097	-	-	-	-	-	-	-	-	9
	Codium setchellii	no	n.s.	8.65 (0.03)	6	-14.52	1	0.07 (0.02)	**0.030**	2229.00 (21.00)	6	31.33 (30.06)	0.214	8.68 (0.36)	5.51 (0.23)	3.21 (0.11)	2.04 (0.07)	11
	Ulva intestinalis	yes	decrease	10.08 (0.06)	6	-15.20 (1.06)	8	-0.35 (0.12)	**0.030**	1354.20 (47.34)	5	-764.30 (57.85)	**< 0.001**	9.25 (0.65)	5.82 (0.41)	2.64 (0.07)	1.66 (0.04)	10
	Ulva lactuta	yes	decrease	10.16 (0.06)	10	-12.49 (2.16)	12	-0.13 (0.04)	**0.019**	1540.00 (106.42)	4	-687.25 (113.26)	**0.007**	11.35 (1.31)	7.20 (0.83)	3.03 (0.19)	1.92 (0.12)	4, 14
	Urospora sp.	yes	decrease	10.23 (0.02)	3	-16.51	1	-0.32 (0.06)	**0.035**	1275.67 (68.67)	3	-922.00 (77.73)	**0.005**	7.82 (0.88)	4.96 (0.56)	3.21 (0.11)	2.04 (0.07)	11
Phaeophyta	*Alaria marginata*	yes	decrease	9.42 (0.01)	6	-13.01 (0.46)	5	0.13 (0.07)	0.115	2160.00 (27.69)	5	-103.83 (53.50)	**0.018**	15.47 (0.24)	9.81 (0.15)	2.75 (0.07)	1.74 (0.04)	12
	Analipus japonicus	yes	-	9.48 (0.04)	6	-16.37	1	0.10 (0.03)	**0.013**	-	-	-	-	-	-	-	-	5, 6
	Fucus gardneri	yes	n.s.	9.87 (0.01)	10	-14.37	1	-0.05 (0.04)	0.232	1881.50 (221.93)	4	-394.00 (229.53)	0.174	14.90 (2.37)	9.47 (1.51)	2.57 (0.07)	1.64 (0.04)	4, 13
	Leathesia marina	yes	-	9.21 (0.05)	6	-14.21	1	0.23 (0.02)	**< 0.001**	-	-	-	-	-	-	-	-	8
	Pelvetiopsis limitata	yes	-	9.55 (0.05)	6	-17.18	1	0.14 (0.02)	**< 0.001**	-	-	-	-	-	-	-	-	5, 6
	Saccharina groendlandica	yes	n.s.	9.08 (0.07)	12	-23.11	1	0.21 (0.04)	**< 0.001**	2229.17 (17.37)	6	-34.67 (23.08)	0.106	14.05 (0.33)	8.92 (0.21)	2.75 (0.07)	1.74 (0.04)	8, 12
	Saccharina sessilis	no	-	8.96 (0.11)	5	-16.67	1	-0.09 (0.09)	0.404	-	-	-	-	-	-	-	-	9
Rhodophyta	*Bossiella sp.*†	yes	-	9.43 (0.03)	6	-15.33	1	-0.07 (0.04)	0.144	-	-	-	-	-	-	-	-	7
	Callithamnion pikeanum	no	n.s.	8.78 (0.07)	7	-31.1	1	0.04 (0.02)	0.141	2221.67 (116.36)	3	24.00 (125.42)	0.856	6.74 (0.48)	4.28 (0.31)	3.21 (0.11)	2.04 (0.07)	8, 11
	Corallina frondescens†	yes	decrease	9.59 (0.03)	5	-20.46	1	0.06	-	1105.60 (33.23)	5	-1169.90 (40.07)	**< 0.001**	6.96 (0.35)	4.42 (0.22)	2.57 (0.07)	1.64 (0.04)	13, 14
	Corallina vancouveriensis†	yes	decrease	9.34 (0.02)	6	-5.69	1	-0.07 (0.03)	0.086	1228.17 (23.18)	6	-1035.67 (28.89)	**< 0.001**	6.88 (0.36)	4.37 (0.23)	2.75 (0.07)	1.74 (0.04)	12
	Cryptopleura ruprechtiana	no	decrease	8.64 (0.06)	10	-33.49	1	0.09 (0.01)	**< 0.001**	1963.00 (28.68)	3	-34.67 (23.08)	**0.002**	4.32 (0.24)	2.74 (0.15)	3.03 (0.19)	1.92 (0.12)	8, 14
	Cumagloia andersonii	yes	-	9.77 (0.10)	6	-16.23	1	0.21 (0.03)	**< 0.001**	-	-	-	-	-	-	-	-	5, 6
	Dilsea pygmaea	yes	n.s.	9.32 (0.01)	2	-16.85	1	0.20 (0.08)	0.228	2071.00 (210.00)	2	-126.67 (219.06)	0.654	15.08 (2.08)	9.57 (1.32)	3.21 (0.11)	2.04 (0.07)	11
	Endocladia muricata	yes	decrease	9.22 (0.01)	6	-18.43	1	0.04 (0.06)	0.592	2132.17 (14.29)	6	-65.50 (23.35)	**0.004**	14.12 (0.25)	8.96 (0.16)	3.21 (0.11)	2.04 (0.07)	11
	Halosaccion glandiformis	yes	decrease	10.39 (0.01)	6	-14.95	1	0.09 (0.06)	0.202	2030.67 (39.45)	6	-244.83 (47.05)	**0.001**	14.63 (0.50)	9.30 (0.32)	2.57 (0.07)	1.64 (0.04)	13
	Hymenena multiloba	no	decrease	8.83 (0.01)	10	-31.77	1	0.02 (0.06)	0.696	2031.25 (44.48)	4	-244.25 (51.32)	**0.020**	8.37 (0.43)	5.31 (0.27)	3.03 (0.19)	1.92 (0.12)	4, 14
	Lithothamnion phymatodeum‡	no	decrease	8.64 (0.03)	10	-	-	0.00 (0.01)	0.803	2089.25 (30.86)	4	-186.25 (38.46)	**0.007**	7.57 (0.40)	4.81 (0.51)	2.57 (0.07)	1.64 (0.04)	10, 13
	Mastocarpus alaskensis	yes	n.s.	9.95 (0.05)	10	-14.11	1	-0.11 (0.05)	0.069	2114.75 (111.21)	4	-112.50 (118.05)	0.387	17.40 (1.02)	11.04 (0.65)	3.03 (0.19)	1.92 (0.12)	7, 14
	Mastocarpus jardiini	yes	-	9.46 (0.20)	6	-16.95	1	0.13 (0.05)	0.059	-	-	-	-	-	-	-	-	9
	Mazzaella affinis	yes	-	9.43 (0.03)	6	-20.69	1	0.07 (0.03)	0.092	-	-	-	-	-	-	-	-	9
	Mazzaella flaccida	yes	-	9.49 (0.00)	6	-17.76 (1.25)	2	0.27 (0.04)	**< 0.001**	-	-	-	-	-	-	-	-	5, 6
	Mazzaella parksii	yes	-	9.31 (0.02)	6	-22.72	1	0.10 (0.04)	0.054	-	-	-	-	-	-	-	-	7
	Mazzaella splendens	yes	-	9.29 (0.07)	6	-18.37	1	0.03 (0.02)	0.165	-	-	-	-	-	-	-	-	9
	Microcladia borealis	yes	-	9.36 (0.02)	6	-20.22	1	0.21 (0.03)	**0.001**	-	-	-	-	-	-	-	-	8
	Neorhodomela larix	yes	n.s.	9.50 (0.03)	6	-20.58 (0.34)	5	-	-	2388.00 (104.96)	4	160.75 (111.80)	0.223	19.37 (1.01)	12.29 (0.64)	3.03 (0.19)	1.92 (0.12)	14
	Odonthalia floccosa	yes	decrease	9.39 (0.01)	4	-	-	0.13 (0.06)	0.072	2054.83 (63.40)	6	-209.00 (69.11)	**0.021**	14.70 (0.49)	9.33 (0.31)	2.75 (0.07)	1.74 (0.04)	12
	Osmundea spectabilis	yes	-	9.36 (0.04)	6	-16.31	1	0.28 (0.03)	**< 0.001**	-	-	-	-	-	-	-	-	5, 6
	Palmaria palmata	yes	-	9.26 (0.02)	6	-18.52 (0.74)	27	0.09 (0.07)	0.216	-	-	-	-	-	-	-	-	7
	Porphyra sp.	yes	increase	9.62 (0.05)	10	-20.74	1	0.24 (0.01)	**< 0.001**	2482.00 (47.87)	4	254.75 (54.71)	**0.012**	21.07 (0.69)	13.37 (0.44)	3.03 (0.19)	1.92 (0.12)	4, 14
	Pseudolithophyllum whidbeyense‡	no	decrease	8.48 (0.05)	5	-	-	0.01 (0.01)	0.271	2138.80 (23.61)	5	-136.70 (31.21)	**0.003**	5.03 (0.28)	3.20 (0.18)	2.57 (0.07)	1.64 (0.04)	13
	Weeksia coccinea	yes	n.s.	9.18 (0.01)	6	-20.27	1	0.02 (0.03)	0.542	2335.67 (35.07)	6	60.17 (42.67)	0.149	11.25 (1.21)	7.16 (0.77)	2.57 (0.07)	1.64 (0.04)	11, 13
Viridiplante	*Phyllospadix scouleri*	yes	decrease	9.76 (0.03)	6	-14.91 (0.88)	3	0.04 (0.07)	0.633	1049.67 (97.80)	6	-1214.17 (103.51)	**< 0.001**	6.49 (0.99)	4.12 (0.63)	2.75 (0.07)	1.74 (0.04)	12
