# Supplementary material for: Functional Traits for Carbon Access in Macrophytes
Source: PLoS One. 2016 Jul 14;11(7):e0159062. doi: 10.1371/journal.pone.0159062 (PMC4944969; doi:10.1371/journal.pone.0159062)
Supplement: S3 Table — Species data are compiled and averaged by family membership into 30 families for ancestral state reconstruction and stochastic character mapping. Mean species pH* are taken from a meta-analysis of 25 pH* studies (Stepien 2015). † indicates data from this study contributed to the species mean. Families in which at least one member has a CCM were designated as having CCMs. Grey shading indicates families and species that are categorized as having CCMs. The cutoff for CCM presence in individual species was pH* > 9.05. (PDF) [file pone.0159062.s006.pdf]

S3 Table. Mean pH* and δ^{13}C for 111 species from Phylum Rhodophyta. Species data are compiled and averaged by family membership into 30 families for ancestral state reconstruction and stochastic character mapping. Mean species pH* and δ^{13}C are taken from a meta analysis of 76 studies [13]. † indicates data from this study contributed to the species means. * indicates that the value ± standard error is not significantly different from a cutoff point, and thus is ruled as not having a CCM. Families in which at least one member has a CCM were designated as having CCMs. Grey shading indicates families, species and data that are categorized as having CCMs based on the given metric. The cutoff for CCM presence in individual species was pH* > 9.05 and/or δ^{13}C > -30‰. Missing data are indicated by a dash (-). Ambiguous pH* data (8.90-9.05) that lacked δ^{13}C data were excluded from the dataset (line strikethrough).

family	CCM in at least one family member	Mean family pH*	Total n	% of species with CCM	% of species without CCM	species	mean species pH*	mean species δ^{13}C (‰)	species CCM by pH* / δ^{13}C
Balliaceae	no	8.76	1	-	-	*Ballia callitricha*	8.76	-32.86	no / no
Bangiaceae	yes	9.63	10	90%	10%	*Porphyra umbilicalis*	9.91	-20.20	yes / yes
						Porphyra perforata	9.84	-15.96	yes / yes
						Porphyra fucicola	9.80	-	yes/ -
						Porphyra endiviifolium	9.77	-24.13	yes / yes
						Polysiphonia nigrescens	9.70	-14.47	yes / yes
						Porphyra papenfussii	9.69	-	yes/ -
						Porphyra sp. 3 †	9.62	-	yes/ -
						Polysiphonia fucoides	9.60	-14.47	yes / yes
						Porphyra torta	9.55	-	yes/ -
						Polysiphonia sp. 4	8.78	-33.21	no / no
Bonnemaisoniaceae	no	8.89	1	-	-	*Bonnemaisonia nootkana*	8.89	-	no/ -
Ceramiaceae_Call	yes	9.03	2	50%	50%	*Callithamnion pikeanum* †	9.21	-31.11	yes / no
						Euptilota articulata	8.84	-31.96	no / no
Ceramiaceae_CerAnt	yes	9.86	3	100%	0%	*Ceramium pacificum*	10.25	-	/ -yes
						Ceramium rubrum	9.72	-18.06	yes / yes
						Microcladia borealis †	9.60	-20.22	yes / yes
						~~*Pterothamnion pectinatum*~~	~~8.97~~	~~-~~	~~no/ -~~
Ceramiaceae_Grif	no	8.96	2	0%	100%	*Anotrichium crinitum*	8.98	-30.99	no/ no
						Ptilota plumosa	8.94	-33.66	no/ no
Champiaceae	yes	9.59	1	-	-	*Neogastroclonium subarticulatum*	9.59	-	yes/ -

Continued on next page

S3 Table continued. Mean pH* and $\delta^{13}C$ for each of 111 species from Phylum Rhodophyta.

family	CCM in at least one family member	Mean family pH*	Total n	% of species with CCM	% of species without CCM	species	mean species pH*	mean species $\delta^{13}C$ (‰)	species CCM by pH* / $\delta^{13}C$
Corallinaceae	yes	9.05	8	63%	37%	*Corallina frondescens* †	9.59	-20.46	yes / yes
						Bossiella sp. 1 †	9.43	-15.33	yes / yes
						Corallina vancouveriensis †	9.34	-5.69	yes / yes
						Corallina officinalis	9.17	-13.85	yes / yes
						Jania rosea	8.92	-20.43	no / yes
						Arthrocardia sp. 1	8.86	-	no / -
						Lithothamnion phymatodeum †	8.64	-	no/ -
						Pseudolithophyllum whidbeyense †	8.48	-	no/ -
Cystocloniaceae	yes	8.92	3	33%	67%	*Cystoclonium purpureum*	9.40	-13.88	yes / yes
						Craspedocarpus venosus	8.79	-35.05	no / no
						Rhodophyllis membranacea	8.59	-31.75	no / no
Delesseriaceae_Delles	no	8.75	3	0%	100%	*Membranoptera alata*	8.79	-32.17	no / no
						Hemineura frondosa	8.76	-32.54	no / no
						Delesseria sanguinea	8.70	-33.51	no / no
Delesseriaceae_Phyco	no	8.75	8	0%	100%	~~*Polyneura latissima*~~	~~8.98~~	~~-~~	~~no/ -~~
						Hymenena flabelligera	8.89	-	no/ -
						Hymenena durvilleai	8.85	-30.93	no / no
						Hymenena multiloba †	8.83	-31.77	no / no
						Myriogramme gunniana	8.79	-35.45	no / no
						Halicnide similans	8.74	-33.95	no / no
						Phycodrys rubens	8.70	-33.94	no / no
						Cryptopleura ruprechtiana †	8.64	-33.49	no / no
						Schizoseris sp. 1	8.59	-	no/ -
Dumontiaceae	yes	9.57	6	100%	0%	*Dumontia contorta*	10.00	-14.40	yes / yes
						Dumontia incrassata	9.85	-	yes/ -
						Constantinea subulifera	9.70	-	yes/ -

Continued on next page

S3 Table continued. Mean pH* and $\delta^{13}C$ for each of 111 species from Phylum Rhodophyta.

family	CCM in at least one family member	Mean family pH*	Total n	% of species with CCM	% of species without CCM	species	mean species pH*	mean species $\delta^{13}C$ (‰)	species CCM by pH* / $\delta^{13}C$
						Dilsea carnosa	9.40	-19.70	yes / yes
						Dilsea pygmaea †	9.32	-16.85	yes / yes
						Weeksia coccinea †	9.18	-20.27	yes / yes
Endocladiaceae	yes	9.43	1	-	-	*Endocladia muricata* †	9.43	-18.43	yes / yes
Fryeellaceae	no	8.85	1	-	-	*Fryeella gardneri*	8.85	-	no/ -
Furcellariaceae	yes	9.12	2	50%	50%	*Furcellaria lumbricalis*	9.20	-20.99	yes / yes
						Opuntiella californica	9.04	-33.33	no / no
Gigartinaceae	yes	9.49	7	100%	0%	*Chondracanthus exasperatus*	9.71	-	yes/ -
						Iridaea cordata	9.60	-17.00	yes / yes
						Chondrus crispus	9.56	-19.83	yes / yes
						Mazzaella flaccida †	9.49	-17.76	yes / yes
						Mazzaella affinis †	9.43	-20.69	yes / yes
						Mazzaella splendens †	9.36	-18.37	yes / yes
						Mazzaella parksii †	9.31	-22.72	yes / yes
Gracilariaceae	yes	9.30	6	83%	17%	*Gracilariopsis lemaneiformis*	9.58	-	yes/ -
						Gracilaria conferta	9.50	-	yes/ -
						Gracilaria pacifica	9.43	-	yes/ -
						Gracilaria gaditana	9.40	-	yes/ -
						Gracilaria sp. 3	9.06	-17.22	yes / yes
						Curdiea angustata	8.81	-32.88	no / no
Halymeniaceae	yes	9.14	7	57%	43%	*Prionitis sternbergii*	9.55	-	yes/ -
						Prionitis lanceolata	9.46	-	yes/ -
						Halymenia schizymenioides	9.38	-	yes/ -
						Halymenia gardneri	9.35	-	yes/ -
						~~*Polyopes constrictus*~~	~~8.96~~	~~-~~	~~no/ -~~
						Halymenia sp. 2	8.94	-35.16	no / no
						Thamnoclonium dichotomum	8.79	-30.13	no / no

Continued on next page

S3 Table continued. Mean pH* and δ^{13}C for each of 111 species from Phylum Rhodophyta.

**family**	**CCM in at least one family member**	**Mean family pH***	**Total n**	**% of species with CCM**	**% of species without CCM**	**species**	**mean species pH***	**mean species δ^{13}C (‰)**	**species CCM by pH* / δ^{13}C**
						Carpopeltis phyllophora	8.49	-	no/ -
Kallymeniaceae	no	8.81	4	0%	100%	*Euthora cristata*	8.92	-	no/ -
						Callophyllis rangiferina	8.82	-33.80	no / no
						Kallymenia cribrosa	8.81	-	no/ -
						Callophyllis lambertii	8.71	-33.64	no / no
Liagoraceae	yes	9.77	1	-	-	*Cumagloia andersonii* †	9.77	-16.23	yes / yes
Lomentariaceae	no	8.98	1	-	-	*Lomentaria articulata*	8.98	-29.32*	no / no
Palmariaceae	yes	10.32	3	100%	0%	*Palmaria decipiens*	10.61	-17.56	yes / yes
						Halosaccion glandiforme †	10.47	-14.95	yes / yes
						Palmaria palmata †	9.87	-18.52	yes / yes
Peyssonneliaceae	yes	8.88	1	-	-	*Sonderopelta coriacea*	8.88	-18.32	no / yes
Phacelocarpaceae	no	8.82	1	-	-	*Phacelocarpus peperocarpos*	8.82	-33.21	no / no
Phyllophoraceae	yes	9.84	5	100%	0%	*Mastocarpus papillatus*	10.34	-	yes
						Mastocarpus alaskensis †	9.95	-14.11	yes / yes
						Mastocarpus stellatus	9.90	-19.13	yes / yes
						Ahnfeltiopsis leptophylla	9.56	-	yes / -
						Mastocarpus jardiini †	9.46	-16.95	yes / yes
Plocamiaceae	no	8.69	3	0%	100%	*Plocamium cartilagineum*	8.87	-32.94	no / no
						Plocamium dilatatum	8.64	-33.50	no / no
						Plocamium sp. 1	8.56	-32.50	no / no
Rhodomelaceae	yes	9.52	10	100%	0%	*Neorhodomela oregona*	10.19	-	yes / -
						Neorhodomela larix †	9.99	-20.58	yes / yes
						Odonthalia floccosa †	9.80	-	yes / -
						Polysiphonia lanosa	9.77	-20.70	yes / yes
						Osmundea pinnatifida	9.73	-15.61	yes / yes
						Polysiphonia hendryi gardneri	9.54	-	yes / -
						Osmundea spectabilis †	9.48	-16.31	yes / yes

Continued on next page

S3 Table continued. Mean pH* and $\delta^{13}C$ for each of 111 species from Phylum Rhodophyta.

**family**	**CCM in at least one family member**	**Mean family pH***	**Total n**	**% of species with CCM**	**% of species without CCM**	**species**	**mean species pH***	**mean species $\delta^{13}C$ (‰)**	**species CCM by pH* / $\delta^{13}C$**
						Odonthalia washingtoniensis	9.24	-	yes / -
						Lenormandia marginata	9.01	-23.25	no / yes
						Laurencia sp. 2	8.40	-11.00	no / yes
Rhodymeniaceae	no	8.71	2	0%	100%	~~*Sparlingia pertusa*~~	~~8.97~~	~~-~~	~~no / -~~
						Rhodymenia sp. 2	8.69	-33.27	no / no
						Gloiosaccion brownii	8.48	-33.99	no / no
Schizymeniaceae	yes	9.02	1	-	-	*Schizymenia pacifica*	9.02	-18.46	no / yes
Solieriaceae	yes	9.23	2	100%	0%	*Sarcodiotheca gaudichaudii*	9.32	-	yes / -
						Sarcodiotheca furcata	9.13	-	yes / -
