# Supplementary material for: Functional Traits for Carbon Access in Macrophytes
Source: PLoS One. 2016 Jul 14;11(7):e0159062. doi: 10.1371/journal.pone.0159062 (PMC4944969; doi:10.1371/journal.pone.0159062)
Supplement: S4 Table — Species data are compiled and averaged by family membership into 18 families for ancestral state reconstruction and stochastic character mapping. Mean species pH* are taken from a meta analysis of 25 pH* studies (Stepien 2015). † indicates data from this study contributed to the species mean. Families in which at least one member has a CCM were designated as having CCMs. Grey shading indicates families and species that are categorized as having CCMs. The cutoff for CCM presence in individual species was pH* > 9.05. (PDF) [file pone.0159062.s007.pdf]

S4 Table. Mean pH* and $\delta^{13}C$ for 51 species from Phylum Ochrophyta. Species data are compiled and averaged by family membership into 18 families. Mean species pH* and $\delta^{13}C$ are taken from a meta analysis of 76 studies [13]. † indicates data from this study contributed to the species mean. Families in which at least one member has a CCM were designated as having CCMs. Grey shading indicates families, species and data that are categorized as having CCMs based on the given metric. The cutoff for CCM presence in individual species was pH* > 9.05 and/or $\delta^{13}C$ > -30‰. Missing data are indicated by a dash (-). Ambiguous pH* data (8.90-9.05) that lacked $\delta^{13}C$ data were excluded from the dataset (line strikethrough).

family	CCM in at least one family member	mean family pH*	Total n	% species with CCM	% species without CCM	species	mean species pH*	mean species $\delta^{13}C$ (‰)	species CCM by pH* / $\delta^{13}C$
Alariaceae	yes	9.23	3	100%	0%	*Alaria marginata* †	9.42	-13.01	yes / yes
						Alaria esculenta	9.33	-18.16	yes / yes
						Undaria pinnatifida	9.09	-19.60	yes / yes
Chordaceae	yes	9.20	1	-	-	*Chorda filum*	9.20	-16.22	yes / yes
Chordariaceae	yes	9.68	2	100%	0%	*Leathesia difformis*	10.15	-14.16	yes / yes
						Leathesia marina †	9.21	-14.21	yes / yes
Desmarestiaceae	yes	8.98	1	-	-	*Desmarestia aculeata*	8.98	-22.58	no / yes
Dictyotaceae	yes	9.01	3	25%	75%	*Dictyopteris muelleri*	9.27	-19.08	yes / yes
						~~*Dictyota sp. 2*~~	~~8.99~~	~~-~~	~~no / -~~
						Zonaria angustata	8.94	-21.81	no / yes
						Zonaria turneriana	8.82	-19.73	no / yes
Fucaceae	yes	9.77	7	100%	0%	*Fucus vesiculosus*	10.03	-16.77	yes / yes
						Fucus gardneri †	9.87	-14.37	yes / yes
						Fucus spiralis	9.84	-17.08	yes / yes
						Ascophyllum nodosum	9.72	-17.51	yes / yes
						Fucus serratus	9.70	-17.19	yes / yes
						Pelvetia canaliculata	9.70	-19.14	yes / yes
						Pelvetiopsis limitata †	9.55	-17.18	yes / yes
Himanthaliaceae	yes	9.89	1	-	-	*Himanthalia elongata*	9.89	-14.58	yes / yes
Hormosiraceae	yes	9.77	1	-	-	*Hormosira banksii*	9.77	-11.68	yes / yes
Laminariaceae	yes	9.14	6	100%	0%	*Saccharina latissima*	9.38	-17.28	yes / yes
						Laminaria digitata	9.21	-16.23	yes / yes
						Macrocystis pyrifera	9.14	-17.14	yes / yes

Continued on next page

S4 Table continued. Mean pH* and $\delta^{13}C$ for each of 51 species from Phylum Ochrophyta.

family	CCM in at least one family member	Mean family pH*	Total n	% species with CCM	% species without CCM	species	mean species pH*	mean species $\delta^{13}C$ (‰)	species CCM by pH* / $\delta^{13}C$
						Laminaria hyperborea	9.10	-18.21	yes / yes
						Saccharina groendlandica †	9.08	-23.11	yes / yes
						Saccharina sessilis †	8.96	-16.67	no / yes
Lessoniaceae	yes	8.97	1	-	-	*Ecklonia radiata*	8.97	-18.84	no / yes
Notheiaceae	yes	9.85	1	-	-	*notheia anomala*	9.85	-13.88	yes / yes
Ralfsiaceae	yes	9.48	1	-	-	*Analipus japonicus* †	9.48	-16.37	yes / yes
Sargassaceae	yes	9.27	12	100%	0%	*Sargassum muticum*	9.74	-19.67	yes / yes
						Halidrys siliquosa	9.50	-21.05	yes / yes
						Sargassum lacerifolium	9.38	-18.72	yes / yes
						Sargassum henslowianum	9.37	-	yes / -
						Cystophora retroflexa	9.23	-12.24	yes / yes
						Landsburgia quercifolia	9.20	-18.78	yes / yes
						Carpophyllum flexuosum	9.20	-15.90	yes / yes
						Carpophyllum plumosum	9.17	-	yes / -
						Carpophyllum maschalocarpum	9.15	-	yes / -
						Carpoglossum confluens	9.14	-	yes / -
						Carpophyllum angustifolium	9.14	-	yes / -
						Sargassum heteromorphum	9.08	-18.77	yes / yes
Scytosiphonaceae	yes	8.94	1	-	-	*Colpomenia sinuosa*	8.94	-13.26	no / yes
Seirococcaceae	yes	9.09	3	100%	0%	*Seirococcus axillaris*	9.19	-17.57	yes / yes
						Phyllospora comosa	9.09	-18.69	yes / yes
						Marginariella boryana	8.99	-18.07	no / yes
Sporochnaceae	yes	9.35	1	-	-	*Carpomitra costata*	9.35	-22.54	yes / yes
						~~*Sporochnus sp. 1*~~	~~8.90~~	~~-~~	~~no / -~~
Stypocaulaceae	yes	9.24	2	100%	0%	*Halopteris sp. 1*	9.25	-	yes / -
						Halopteris paniculata	9.23	-22.82	yes / yes
Xiphophoraceae	yes	9.39	2	100%	0%	*Xiphophora chondrophylla*	9.45	-14.79	yes / yes
						Xiphophora gladiata	9.34	-18.95	yes / yes
