# Supplementary material for: Functional Traits for Carbon Access in Macrophytes
Source: PLoS One. 2016 Jul 14;11(7):e0159062. doi: 10.1371/journal.pone.0159062 (PMC4944969; doi:10.1371/journal.pone.0159062)
Supplement: S5 Table — ΔDIC is the deviation of Dissolved Inorganic Carbon concentration from expected DIC depletion under control Total Alkalinity conditions. ΔHCO3- and ΔCO2 are carbon gained due to TA-induced pH shifts driven by macroalgae. N indicates number of individuals per species. p-value is for a two-tailed t-test for changes in carbon concentrations. † indicates articulated calcifying species. ‡ indicates crust-forming calcifying species. Bolding indicates p-value less than 0.050. (PDF) [file pone.0159062.s008.pdf]

S5 Table. **Changes in seawater carbon concentrations after incubation with 23 species of macroalgae and 1 species of surfgrass.** ΔDIC is deviation of Dissolved Inorganic Carbon concentration from expected DIC depletion under control Total Alkalinity conditions. ΔHCO_3^{2-} and ΔCO_2 are carbon gained due to TA-induced pH shifts driven by macroalgae. N indicates number of individuals per species. p value is for a two-tailed t test for changes in carbon concentrations. † indicates articulated calcifying species. ‡ indicates crust-forming calcifying species. Bolding indicates p value less than 0.050.

Division	Taxa	N	Δ DIC (SEM) µmol/kgSW	p ΔDIC	ΔHCO_3^{2-} (SEM) µmol/kgSW	p ΔHCO_3^{2-}	ΔCO_2 (SEM) µmol/kgSW	p ΔCO_2
Chlorophyta	*Acrosiphonia coalita*	4	-458.46 (65.54)	**< 0.001**	79.99 (11.53)	**0.040**	0.02 ($4.2x10^{-3}$)	0.154
	Codium setchellii	6	-1.06 (17.29)	0.284	-22.28 (6.21)	0.203	-0.34 (0.08)	0.157
	Ulva intestinalis	5	-462.32 (25.05)	**< 0.001**	148.32 (60.11)	0.332	0.17 (0.08)	0.367
	Ulva lactuta	4	-393.06 (54.99)	**0.007**	97.01 (16.73)	0.065	0.02 (0.01)	0.190
	Urospora sp.	3	-523.74 (37.96)	**0.007**	31.28 (3.42)	**0.034**	$2.9x10^{-3}$ ($4.5x10^{-4}$)	0.066
Phaeophyta	*Alaria marginata*	5	-45.67 (18.24)	**0.012**	76.01 (9.04)	**0.020**	0.09 (0.01)	**0.021**
	Fucus gardneri	4	-238.41 (132.44)	0.170	84.32 (45.52)	0.423	0.01 (0.02)	0.893
	Saccharina groendlandica	6	-0.85 (12.35)	0.106	26.19 (5.39)	0.104	0.07 (0.01)	0.091
Rhodophyta	*Callithamnion pikeanum*	3	-5.26 (99.82)	0.864	-12.14 (46.57)	0.894	-2.54 (1.37)	0.400
	Corallina frondescens†	5	-711.21 (31.57)	**< 0.001**	152.71 (7.62)	**< 0.001**	0.06 (0.01)	**0.010**
	Corallina vancouveriensis†	6	-710.97 (27.99)	**< 0.001**	357.59 (7.59)	**< 0.001**	0.33 (0.02)	**0.002**
	Cryptopleura ruprechtiana	3	-250.67 (35.25)	**0.022**	187.37 (15.40)	**0.020**	4.73 (0.28)	**0.011**
	Dilsea pygmaea	2	-105.27 (138.03)	0.671	83.18 (102.84)	0.669	0.06 (0.10)	0.766
	Endocladia muricata	6	-67.37 (9.97)	**0.008**	49.41 (4.36)	**0.006**	0.10 (0.01)	**0.006**
	Halosaccion glandiformis	6	-129.96 (20.95)	0.001	14.29 (0.85)	**< 0.001**	$1.0x10^{-3}$ ($7.2x10^{-5}$)	**0.002**
	Hymenena multiloba	4	-161.70 (36.20)	**0.026**	143.43 (16.34)	**0.022**	1.04 (0.12)	**0.023**
	Lithothamnion phymatodeum‡	4	-155.43 (25.19)	**0.005**	134.24 (11.29)	**0.009**	1.41 (0.11)	**0.007**
	Mastocarpus alaskensis	4	-63.42 (63.67)	0.377	15.60 (25.07)	0.776	-0.02 (0.02)	0.680
	Neorhodomela larix	4	97.13 (64.44)	0.232	-122.07 (40.48)	0.229	-0.18 (0.07)	0.281
	Odonthalia floccosa	6	-114.65 (41.39)	**0.019**	144.05 (17.53)	**0.020**	0.13 (0.02)	**0.038**
	Porphyra sp.	4	149.00 (27.61)	**0.017**	-181.85 (17.45)	0.014	-0.17 (0.02)	**0.038**
	Pseudolithophyllum whidbeyense‡	5	-122.87 (20.99)	**0.006**	92.92 (7.50)	**0.005**	2.94 (0.19)	**0.002**
	Weeksia coccinea	6	46.57 (28.48)	0.162	-44.14 (10.42)	0.145	-0.61 (0.20)	0.275
Viridiplante	*Phyllospadix scouleri*	6	-756.59 (72.36)	**< 0.001**	151.92 (4.60)	**< 0.001**	0.07 ($4.8x10^{-3}$)	**0.002**
